# Advances in COVID-19 mRNA vaccine development

**DOI:** 10.1038/s41392-022-00950-y

**Published:** 2022-03-23

**Authors:** Enyue Fang, Xiaohui Liu, Miao Li, Zelun Zhang, Lifang Song, Baiyu Zhu, Xiaohong Wu, Jingjing Liu, Danhua Zhao, Yuhua Li

**Affiliations:** 1 National Institute for Food and Drug Control, Beijing, 102629 China; 2 Wuhan Institute of Biological Products, Co., Ltd., Wuhan, 430207 China; 3 Texas A&M University, College Station, TX 77843 USA

**Keywords:** Vaccines, Infectious diseases, Gene delivery

## Abstract

To date, the coronavirus disease 2019 (COVID-19) caused by severe acute respiratory syndrome coronavirus 2 (SARS-CoV-2) has determined 399,600,607 cases and 5,757,562 deaths worldwide. COVID-19 is a serious threat to human health globally. The World Health Organization (WHO) has declared COVID-19 pandemic a major public health emergency. Vaccination is the most effective and economical intervention for controlling the spread of epidemics, and consequently saving lives and protecting the health of the population. Various techniques have been employed in the development of COVID-19 vaccines. Among these, the COVID-19 messenger RNA (mRNA) vaccine has been drawing increasing attention owing to its great application prospects and advantages, which include short development cycle, easy industrialization, simple production process, flexibility to respond to new variants, and the capacity to induce better immune response. This review summarizes current knowledge on the structural characteristics, antigen design strategies, delivery systems, industrialization potential, quality control, latest clinical trials and real-world data of COVID-19 mRNA vaccines as well as mRNA technology. Current challenges and future directions in the development of preventive mRNA vaccines for major infectious diseases are also discussed.

## Introduction

Coronavirus disease 2019 (COVID-19) is an emerging disease caused by severe acute respiratory syndrome coronavirus 2 (SARS-CoV-2).^1–3^ SARS-CoV-2 is an enveloped positive-sense single-stranded RNA (ssRNA) virus of the *Betacoronavirus* genus included in the *Coronaviridae* family. The full-length genome of SARS-CoV-2 isolate Wuhan-Hu-1 consists of 29,881 nucleotides (GenBank accession number: MN908947) with a methylated 5′-cap and a 3′-poly(A) tail, consisting of 9860 amino acids encoding 16 nonstructural proteins (nsp), 9 accessory proteins, and 4 structural proteins. The four structural proteins include spike (S), envelope (E), membrane (M), and nucleocapsid (N) proteins.^4,5^ As of February 9, 2022, COVID-19 has spread to 227 countries and regions worldwide,^6^ causing over 399 million confirmed cases and 5.75 million deaths.^7^ The COVID-19 pandemic is an unprecedented event which has caused a huge impact on human health and global public health security. Currently, no specific drug has been identified for COVID-19 prevention or treatment, and vaccination is the most economical and effective intervention to limit the spread of SARS-CoV-2.

To control the spread of the epidemic, governments worldwide have mobilized a considerable amount of manpower and material resources into research and development (R&D) efforts linked to the COVID-19 vaccine. Several approaches to COVID-19 vaccine development have been tested concurrently, including inactivated-virus,^8–11^ live attenuated,^12–14^ recombinant protein,^15–19^ adenovirus vector,^20–24^ influenza virus vector,^25,26^ mRNA^27–29^ and DNA vaccines.^30–34^ As a revolutionary innovation, the mRNA vaccine technology has played a unique role in controlling the COVID-19 pandemic.

**Fig. 1:**
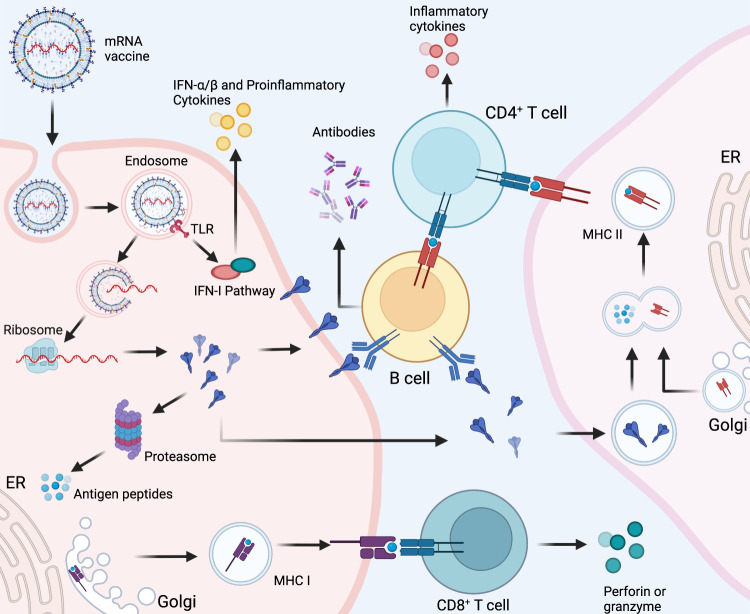
Cellular and humoral immune responses induced by messenger RNA (mRNA) vaccine. mRNA delivered in an mRNA vaccine enters cells by endocytosis and, after release from the endosome, is translated into protein by ribosomes. Translated proteins can then activate the immune system primarily in two ways: i) proteins are degraded by the proteasome into peptides subsequently presented as antigens on the cell surface by major histocompatibility complex (MHC) class I molecules which bind to the T cell receptor (TCR) to activate CD8^+^ T cells to kill infected cells thorugh the secretion of perforin and granzyme; ii) proteins secreted extracellularly are engulfed by antigen-presenting cells (APCs) and degraded into peptides subsequently presented on the cell surface by MHC class II molecules for recognition by CD4^+^ T cells, which can activate both the cellular immune responses by secreting cytokines and the humoral immune responses by co-activating B cells. In addition, single-stranded RNA and double-stranded RNA delivered in mRNA vaccines bind to Toll-like receptor (TLR) in the endosome to activate the antiviral innate immune responses via the production of type-I interferon (IFN-I) which results in the induction of several IFN-1-stimulated genes involved in antiviral innate immunity, in a mechanism known as the self-adjuvant effect of a sequence-engineered mRNA. This figure is created with BioRender.com

**Fig. 2:**
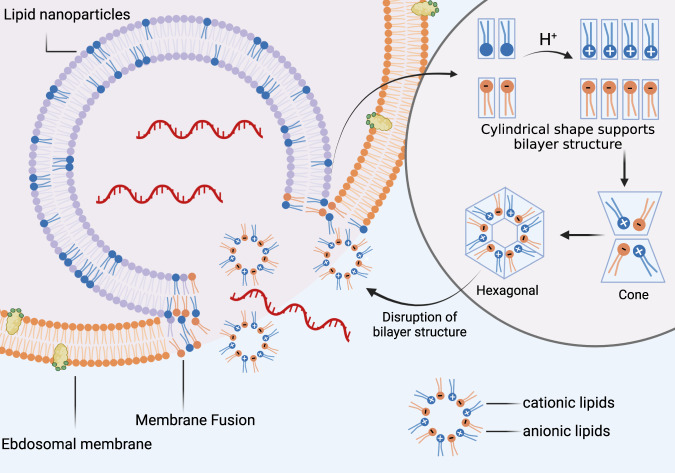
Proposed mechanism of endosomal escape of delivered mRNA. Endosomal escape of delivered mRNA is largely dependable on interactions between ionizable lipids and naturally occurring anionic phospholipids in the endosomal membrane.^43^ Prior to membrane fusion, ionizable lipids in lipid nanoparticles (LNPs) and anionic lipids in the endosomal membrane adopt a cylindrical conformation which is compatible with molecular packing in a bilayer phase. The acidic environment in endosomes facilitates protonation of ionizable lipids into cationic lipids. Cationic and anionic lipids generate ion pairs whose combined cross-sectional headgroup area is smaller than the total of individual headgroup areas before membrane fusion. Consequently, the ion pair adopts a conical shape which promotes the formation of inverted, non-bilayer phases, such as the hexagonal shape illustrated above. Thus, the formation of ion pairs between lipids promotes membrane fusion and disruption, allowing mRNA to escape from endosomes. This figure is created with BioRender.com

The fundamental mechanism underlying the mRNA vaccine technology is based on a vehicle that enables the delivery of a nucleic acid molecule encoding the antigen of interest into the target cell in the human host, thus allowing the host cell to fabricate the target protein and express the antigen to elicit the immune response. In this way, upon invasion by a pathogen carrying the antigen, the immune system of the host can quickly trigger humoral and cellular immune responses, thereby preventing the disease (Fig. 1). Three types of host cells can be transfected after administration of an mRNA vaccine intramuscularly, intracutaneously, or subcutaneously^35^: (1) non-immune cells (such as muscle cells and epidermal cells) at the injection site^36–38^; (2) immune cells found in the tissues at the injection site (such as dendritic cells and macrophages)^39,40^; (3) immune cells in peripheral lymphoid organs after the injected mRNA is transferred through the lymphatic system to adjacent lymph nodes or the spleen.^39,41,42^ Since mRNA is a negatively charged and unstable molecule, it is generally encapsulated in a delivery vehicle in order to enter the target cell. For instance, mRNA delivered by vaccine vehicles based on lipid nanoparticles (LNPs) enters cells exclusively by endocytosis, forming an endosome without destroying the cell membrane. After entering the cytoplasm, the endosome is directed immediately to lysosomes for degradation. Therefore, in order to ensure structural integrity and thus translation of injected mRNA, endosomal fusion with lysosomes and disruption must be evaded. Studies have shown that the ionizable lipids in LNPs play a role in mRNA release and endosomal escape. In the acidic environment inside endosomes, the headgroup of the ionizable lipid is protonated to a cationic state. After attracting and combining with the anionic headgroup of phospholipids in the endosomal membrane, the hydrophobic tail of cationic lipid and phospholipid expands, and the stable phospholipid bilayer structure is disrupted, which in turn allows mRNA to evade the endosome and reach the cytoplasmic compartment (Fig. 2).^35,43–46^ mRNA is then translated into proteins by ribosomes, used as an endogenous antigen, and degraded by the proteasome into antigenic peptides, which are presented to CD8^+^ cytotoxic T cells through the major histocompatibility complex (MHC) class I molecular pathway to activate cell-mediated immune responses, thereby constituting the key advantage of mRNA vaccines. In addition, translated proteins based on the information contained in the mRNA can be secreted into the extracellular environment, thereby entering the circulatory system in which they are uptaken by antigen-presenting cells (APCs). The antigenic peptide is presented to CD4^+^ T cells through MHC class II molecules as an exogenous antigen, which can elicit cellular immune response via the secretion of cytokines and activate B cells to produce antibodies and exert humoral immune effects.^47^ In addition, mRNA vaccines possess a self-adjuvant effect.^48,49^ ssRNA can be recognized by Toll-like receptor 7 (TLR7) and TLR8 in endosomes^50,51^ and activate the myeloid differentiation marker 88 (MyD88) pathway.^52^ Double-stranded (dsRNA) can be recognized by TLR3,^53^ retinoic-acid-inducible gene I protein (RIG-I),^54^ melanoma differentiation-associated gene 5 (MDA5)^55,56^ and other molecules, to cause downstream activation of TIR-domain-containing adapter-inducing interferon-β (TRIF) and mitochondrial antiviral signaling protein (MAVS) molecules,^48,52^ thereby mediating the production of type-I interferons (IFN-I) and pro-inflammatory cytokines^57,58^ as well as activating signaling pathways and several IFN-stimulated genes.^59^ In general, mRNA vaccines induce the production of antibodies, unique cellular immune responses, and self-adjuvant effects by the above-described mechanisms.

The successful development of mRNA vaccines is a result of years of research and groundwork. The mRNA molecule was first described by Brenner and colleagues in 1961,^60^ and due to the highly unstable nature of the mRNA molecule, it was not until 1969 that the first protein was produced in vitro from isolated mRNA.^61^ Dimitriadis and colleagues attempted to employ unilamellar liposome wrapping to deliver exogenous mRNA into human and murine cells in order to avoid mRNA degradation by nucleases.^62,63^ However, RNA is easily degraded and difficult to obtain in vitro, which greatly hinders the progress of research in RNA transfected cells. In 1984, Krieg and colleagues were the first to employ SP6 RNA polymerase to successfully transcribe and synthesize mRNA in vitro, establishing the foundation for subsequent in vitro mRNA studies.^64–74^ Subsequently, in 1987 Malone and colleagues employed cationic lipids to encapsulate mRNA for injection into eukaryotic cells, obtaining a highly efficient system for the expression of mRNA in vitro.^75^ In 1990, Wolff and colleagues injected for the first time into mouse quadriceps muscle mRNA resulted from in vitro transcription (IVT) which was successfully expressed, thus paving the way for mRNA therapeutic research.^76^ After immunizing mice with liposome-encapsulated mRNA encoding the influenza-virus nucleoprotein (NP), the production of anti-influenza cytotoxic T lymphocytes (CTLs) was induced in the host, thus marking a milestone in the development of the first mRNA vaccine.^77^ Later, Conry and colleagues tested the first mRNA tumor vaccine encoding the carcinoembryonic antigen (CEA) in mice, which broadened the perspectives for human anticancer research.^78–80^ However, due to the unsatisfactory stability and safety of mRNA vaccines, their use has been widely disregarded. In 2005, Karikó and colleagues found that mRNA synthesized using modified uridine could avoid recognition and degradation by the immune system, which greatly improved mRNA stability and immunogenicity in vivo, thereby inaugurating a new era in mRNA vaccine.^81^ After years of investigation, in August 2018 the first-ever RNA interference (siRNA) therapeutic drug, Onpattro ® (patisiran) (Alnylam Pharmaceuticals Inc., Cambridge, MA, USA), was approved by the U.S. Food and Drug Administration (FDA).^82–84^ mRNA vaccines for various infectious diseases, such as rabies, influenza, Ebola, Zika, and dengue virus, have entered the preclinical research or clinical trials in recent years.^85–99^ Since the beginning of the COVID-19 pandemic in 2019, mRNA vaccines have become a current research hotspot owing to their shorter R&D cycle, simple production process, and ability to induce strong immune responses.

The mRNA vaccine is the first batch of COVID-19 vaccine candidates in clinical trials. As of February 8, 2022, WHO reported 337 COVID-19 vaccine candidates currently under development, of which 47 are mRNA vaccines, and 23 among these have entered clinical trials.^100^ The mRNA vaccines Pfizer-BioNTech (BNT162b2), Moderna (mRNA-1273), and CureVac^101–103^ were the fastest vaccine development in medical history. The first two obtained emergency use authorization (EUA)^104^ from many regulatory agencies in the United States, the United Kingdom, Canada, and Hong Kong, China. On August 23, 2021, the Pfizer-BioNTech was the first COVID-19 vaccine officially approved for commercialization by the FDA,^105^ being also the first-ever approved on October 29, 2021 for use in children aged 5–11.^106^ Thus, the mRNA vaccine technology has the most promising application prospects for COVID-19. Thus, this review will cover the different types of COVID-19 mRNA vaccines, antigen design strategies, delivery vehicles, clinical trials, production process, and quality control, among other related topics.

## Different Types of COVID-19 mRNA Vaccines

**Fig. 3:**
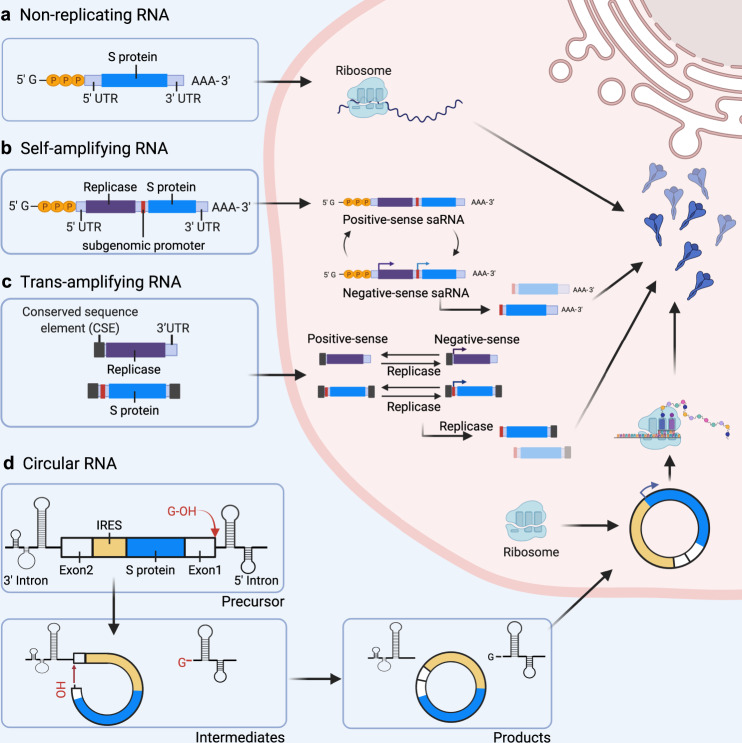
Antigen expression in different types of mRNA vaccines. **A** The vaccine immunogen is encoded by a non-replicating RNA flanked by 5′ and 3′ UTRs (S protein). **B** Self-amplifying RNA (saRNA) encodes four nonstructural proteins (nsp 1–4) and a subgenomic promoter derived from the alphavirus genome. saRNA encodes a replicase and amplifies vaccine-encoding transcripts. **C** Trans-amplifying RNA (taRNA) uses two transcripts to enable self-amplification of replicase and the immunogen. **D** Circular RNA (circRNA) is circularized by the autocatalytic Group I ribozyme.^223^ The exon 2 is ligated upstream to exon 1, and a coding region is inserted between the exon-exon junction. During splicing, the 3′-OH of a guanosine nucleotide engages in a transesterification reaction at the 5′ splice site. The 5′ intron is excised, and the 3′-OH at the end of the intermediate engages in a second transesterification reaction at the 3′ splice site, resulting in the circularization of the immunogen mRNA. Upon entering the cell, the internal ribosome entry site (IRES) of circRNA initiates protein translation. The figures are created with BioRender.com

mRNA vaccines can be categorized as non-replicating mRNA, self-amplifying mRNA (saRNA) and circular RNA (circRNA) based on their genetic characteristics (Fig. 3).^107^ Non-replicating mRNA vaccines deliver exclusively genetic information coding for the target antigen, thus containing the 5′-cap, 5′ untranslated region (UTR), 3′ UTR, and 3′-poly(A) tail regions.^108^ saRNA vaccines can deliver genetic information encoding the target antigen and other genes, e.g., viral RNA polymerase, to enable mRNA to self-replicate.^109,110^ Based on saRNA technology, safe trans-amplifying RNA (taRNA) vaccines have been optimized and developed.^111^ In addition, circRNA has recently been developed for COVID-19 mRNA vaccines due to its natural high stability.^112^ Most COVID-19 mRNA vaccines currently in clinical trials or those already on the market are non-replicating mRNA vaccines. The advantages of non-replicating mRNA vaccines include the use of an RNA molecule of simple structure and shorter length. An optimized or modified mRNA can have greatly enhanced biological activity. Lastly, saRNA vaccines are currently in preclinical and clinical trials.

### Conventional non-replicating mRNA

**Fig. 4:**
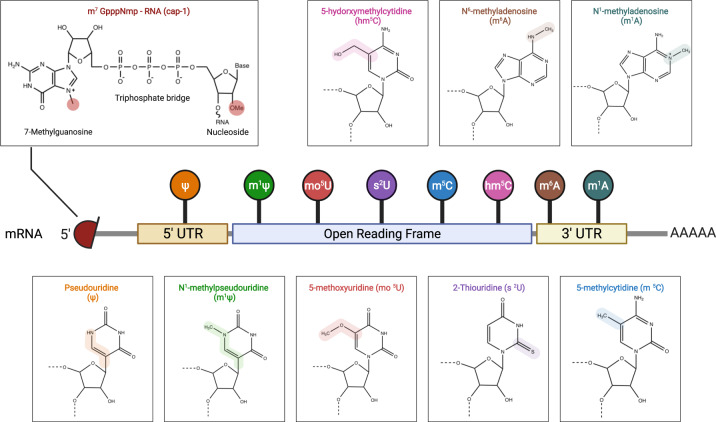
Structure of mRNA and nucleotide modifications. mRNA molecules are synthesized in vitro with a 5′-cap 1 structure and chemically modified nucleotides as substitutes for natural nucleotides, which enhances stability and translation efficiency of mRNA as well as reduces innate immune response. This figure is created with BioRender.com

A non-replicating mRNA contains an open reading frame (ORF) encoding the gene coding for the target antigen flanked by 5′ and 3′ UTR. The 5′ end contains a 7-methylguanosine cap structure (5′-cap, m^7^G), whereas the 3′ end contains a poly(A) tail structure. This structure enhances stability of the delivered mRNA while improving accuracy and efficiency of mRNA translation (Fig. 4).^113,114^

#### 5′-cap

The 5′-cap structure prevents mRNA from degradation by exonucleases, thereby maintaining mRNA stability and enabling translation initiation.^115^ m^7^G is found at the 5′ end of mature mRNA in eukaryotic cells and connected to the first nucleotide of mRNA transcription by triphosphates to form an m^7^G cap structure (m^7^GpppNp).^116^ According to the degree of methylation, three main cap structures are possible: cap 0, cap 1, and cap 2. A cap 0 structure is the most elementary, namely m^7^GpppNp; however, an mRNA of cap 0 is likely to be recognized as exogenous RNA by the host, which could stimulate the innate immune response of the host and ultimately trigger inflammatory responses.^117^ A cap1 structure (m^7^GpppN_1_mp) has a methylated 2′-OH on the first nucleotide connecting the 5′ end of the mRNA to the cap.^118^ Since the cap1 structure has only been described to date in eukaryotic mRNAs, it can be used as a signature of self-RNA, thus reducing the activation of pattern recognition receptor (PRR) and consequently improving translation efficiency of mRNA in vivo.^119^ Lastly, cap2 (m^7^GpppN_1_mpN_2_mp) has a methylated 2′-OH on both the first and second nucleotides that connect the 5′ end of the mRNA to the cap, and methylation improves mRNA translation efficiency.^115^ At present, the cap1 structure is most commonly applied for capping mRNA vaccines.

**Fig. 5:**
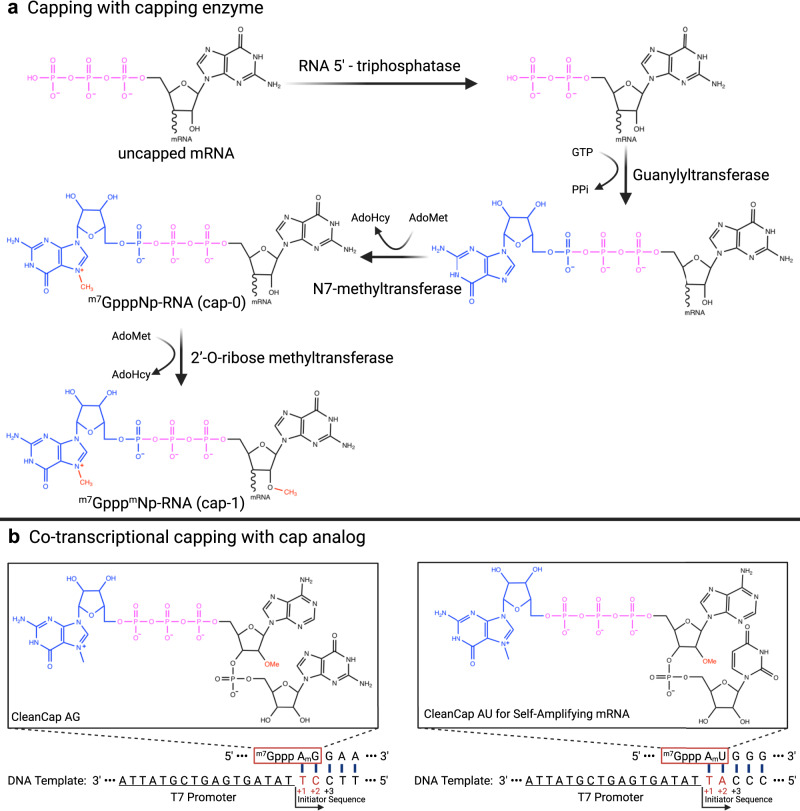
mRNA capping procedure using capping enzymes or cap analogs. **A** Production of post-transcriptional modifications of mRNA with cap0 requires three enzymes: triphosphatase, guanylyltransferase, and N7-methyltransferase with S-adenosylmethionine (SAM) as the methyl donor. Subsequently, the cap0 is modified with 2′-O-ribose methyltransferase to generate the cap1 structure. **B** Cap analogs commonly used for in vitro transcription of mRNA are CleanCap® Reagent AG (TriLink) and CleanCap® Reagent AU (TriLink). The proposed mechanism of CleanCap co-transcriptional initiation involves the docking of AmG or AmU dimers onto the +1 and +2 positions in template nucleotides. Initiation occurs upon coupling of CleanCap with an nucleoside triphosphate (NTP) occupying the +3 position.^157^

Two types of capping methods are possible during IVT of mRNA (Fig. 5). The first method employs a capping enzyme RNA 5′-triphosphatase (RTPase) which hydrolyzes the 5′ γ-phosphate of RNA transcripts, with a transfer of guanosine monophosphate (GMP) to 5′-diphosphate RNA by guanylyltransferase (GTase), and the resulting 5′-end β-phosphate is combined with GMP to form GpppNp-RNA. Finally, the guanosine moiety is methylated by a cap-specific S-adenosylmethionine-(AdoMet)-dependent (guanine-N7) methyltransferase (N7MTase), forming a cap0 structure (m^7^GpppNp). The cap0 structure can be further modified to cap1 (m^7^GpppN_1_mp) by 2′-O-methyltransferase (2′-O-MTase).^120^ The vaccinia capping enzyme (VCE) integrates the enzymatic activity of RTPase, GTase, and G-N7 MTase, which can be capped to generate a cap0 structure, and then 2′-O-MTase can be used to generate a methylated cap1 structure, thus reaching a capping efficiency of 100%.^52^ Of note, it has been reported that the COVID-19 vaccine mRNA-1273 developed by Moderna employs the capping enzyme described above.^28^ The second capping method employs cap analogs (m^7^GpppG) during transcription of mRNA, involving T7, T3, or SP6 RNA polymerases to achieve mRNA co-transcriptional capping.^121^ Co-transcriptional capping is the most commonly used method in IVT for mRNA,^115^ but studies have found that cap analogs bind mRNA strands in reverse orientation.^122^ The reverse-capped mRNA cannot be recognized by the ribosome, resulting in reduced translation efficiency.^123^ To avoid reverse capping, a modified cap analog with methylation modification, namely anti-reverse cap analog (ARCA) (m^7^(3′-O-methyl)-GpppG), has emerged.^124^ Since the 3′-OH group in proximity to m^7^G is methylated, thus the ARCA cap analog can only bind the 5′ end of mRNA in forward orientation, which is recognized by the eukaryotic translation initiation factor 4E (eIF4E) to initiate ribosome recruitment and translation.^74,125^ However, the cap0 structure produced by ARCA capping requires additional methylation modification to yield a stable cap1 structure. Therefore, the ARCA capping approach results in inefficient capping and is not widely adopted. The current new generation of cap analogs is the CleanCap® cap analogs developed by TriLink BioTechnologies (San Diego, CA, USA),^126^ which can be co-transcribed with the target mRNA to obtain the cap1 structure, thus solving the issues of low efficiency and high enzyme costs of traditional capping methods. At present, there are several capping analogs such as CleanCap® Reagent AG (m^7^GpppA^2′OMe^pG), CleanCap® Reagent AU (m^7^GpppA^2′OMe^pU), and CleanCap® Reagent AG 3′OMe (m^7^G^3′OMe^pppA^2′OMe^pG),^127^ among which CleanCap® Reagent AG is commonly used for non-replicating mRNA,^128^ requiring that the T7 promoter sequence at the 5′ end of the DNA template must be followed by an AG start. In contrast, CleanCap® Reagent AU is a capping analog designed specifically for self-replicating RNA,^129^ and the start sequence at the 5′ end of the DNA template must be AU (Fig. 5). The use of CleanCap® cap analogs reduces the probability of reverse capped, uncapped, and cap0 intermediates, and the co-transcribed mRNA only possesses a cap1 structure, therefore capping rate can be 90% or higher.^52,126^ Currently, COVID-19 mRNA vaccines BNT162b1 and BNT162b2 developed by BioNTech employ TriLink Cap1 cap analog (m_2_^7,3′-O^)Gppp(m^2′-O^)ApG for co-transcriptional capping.^27,130^

In summary, cap structure of mRNA can both protect mRNA from destruction and facilitate its recognition by the host due to chemical modifications. Additionally, co-transcriptional capping can increase productivity of mRNA vaccines.

#### 5′ and 3′ UTRs

mRNA contains 5′ and 3′ UTRs, whose functions are related, respectively, to regulating translation and maintaining mRNA stability.^131^ The 5′ UTR is mainly involved in translation of its downstream ORF sequence.^132,133^ The Kozak sequence is generally added after the 5′ UTR sequence to improve translation efficiency.^134^ Conversely, the function of the 3′ UTR is to maintain mRNA stability.^135,136^ Studies have shown that adenylate-uridylate-rich elements are involved in mRNA degradation. Degradation rate and translation life cycle can be adjusted by replacing adenylate-uridylate-rich sequences found in the 3′ UTR.^137–139^ At present, the 3′ UTR is mainly derived from hemoglobin subunit α (*HBA*) and subunit β (*HBB*) genes,^140^ but it can also be derived from albumin (*ALB*), heat-shock protein 70 (*Hsp70*), tyrosine hydroxylase (*TH*), and collagen alpha 1 (*COL1A1*) genes.^141–143^ In contrast, the 5′ UTR is mostly retrieved from genes such as globin, *Hsp70*, axon dynein heavy chain 2 (*DNAH2*), and hydroxysteroid dehydrogenase (*3β*-*HSD*).^144,145^ Design of proper 5′ and 3′ UTRs sequences is crucial for the success of mRNA vaccines. Many investigations have been conducted to screen and design the most effective 5′ and 3′ UTR sequences for mRNA vaccines, therefore UTR sequences are considered intellectual properties of vaccine manufacturers. Table 1 summarizes current UTR sequences of COVID-19 mRNA vaccines from different vaccine manufacturers.

**Table 1:** Design strategies for 5′ and 3′ UTR of mRNA vaccines from different vaccine manufacturers and/or researchers

Vaccine name/Manufacturer	Source	Sequence
BNT162b2/BioNTech^153,476,477^	5′ UTR: Human alpha-globin RNA with an optimized Kozak sequence	GAATAAACTAGTATTCTTCTGGTCCCCACAGACTCAGAGAGAACCCGCCACC
	3′ UTR: The amino-terminal enhancer of split (AES) mRNA and the mitochondrial encoded 12S ribosomal RNA	CTCGAGCTGGTACTGCATGCACGCAATGCTAGCTGCCCCTTTCCCGTCCTGGGTACCCCGAGTCTCCCCCGACCTCGGGTCCCAGGTATGCTCCCACCTCCACCTGCCCCACTCACCACCTCTGCTAGTTCCAGACACCTCCCAAGCACGCAGCAATGCAGCTCAAAACGCTTAGCCTAGCCACACCCCCACGGGAAACAGCAGTGATTAACCTTTAGCAATAAACGAAAGTTTAACTAAGCTATACTAACCCCAGGGTTGGTCAATTTCGTGCCAGCCACACCCTGGAGCTAGC
mRNA1273/ Moderna^153,476^	5′ UTR: NA	GGGAAATAAGAGAGAAAAGAAGAGTAAGAAGAAATATAAGACCCCGGCGCCGCCACC
	3′ UTR: Homo sapiens hemoglobin subunit alpha 1 gene (HBA1)	GCTGGAGCCTCGGTGGCCTAGCTTCTTGCCCCTTGGGCCTCCCCCCAGCCCCTCCTCCCCTTCCTGCACCCGTACCCCCGTGGTCTTTGAATAAAGTCTGAGTGGGCGGCA
CV2CoV/ CureVac^478^	5′ UTR: human hydroxysteroid 17-beta dehydrogenase 4 gene (HSD17B4)	NA
	3′ UTR: human proteasome 20S subunit beta 3 gene (PSMB3)	NA
CVnCoV/ CureVac^478^	5′ UTR: NA	NA
	3′ UTR: parts of the 3′ UTR of the Homo-sapiens alpha hemoglobin gene	NA
LIVERNA^479^	5′ UTR: Dynein Axonemal Heavy Chain 2 (DNAH2)	GAGACCCAAGCTGGCTAGCGGGAGAAAGCTTACCGGCTAGCGCCGCCACC
	3′ UTR: Homo sapiens hemoglobin subunit alpha 2 gene (HBA2)	GCTGGAGCCTCGGTAGCCGTTCCTCCTGCCCGCTGGGCCTCCCAACGGGCCCTCCTCCCCTCCTTGCACCGGCCCTTCCTGGTCTTTGAATAAAGTCTGAGTGGGCAGC
RiboBio^141^	5′ UTR: Homo sapiens hydroxysteroid 17-beta dehydrogenase 4 gene (HSD17B4)	GTCCCGCAGTCGGCGTCCAGCGGCTCTGCTTGTTCGTGTGTGTGTCGTTGCAGGCCTTATTCAGATCTACCGGTGGTACCGCCACC
	3′ UTR: Homo sapiens albumin gene (ALB)	AGCCAACACCCTGTCTAAAAAACATAAATTTCTTTAATCATTTTGCCTCTTTTCTCTGTGCTTCAATTAATAAAAAATGGAAAGAACCT
Stemirna^276^	5′ UTR: NA	GCTCGCTTTCTTGCTGTCCAATTTCTATTAAAGGTTCCTTTGTTCCCTAAGTCCAAGGGGATATTATGAAGGGCCTTGAGCATCTGGATTCTGCCTAATA AAAAACATTTATTTTCATTGC
	3′ UTR: NA	ACATTTGCTTCTGACACAACTGTGTTCACTAGCAACCTCAAACAGACACC

*NA* not applicable; *UTR* untranslated region.

#### Poly(A) tail

The poly(A) tail plays an important role in maintaining mRNA stability and translation efficiency.^116^ mRNA stability can be improved by inhibiting exonuclease-mediated mRNA degradation.^52^ The poly(A) tail can also bind to multiple poly(A)-binding proteins (PABPs) while working synergistically with 5′ m7G cap sequences to regulate translational efficiency.^146,147^ Polyadenylation of engineered mRNA can occur in two ways: i) by traditional enzymatic polyadenylation, adding the poly(A) tail to the 3′ end of mRNA, but which does not allow regulation of tail length^148^; and ii) by designing a fixed-length poly(A) sequence on a DNA template and transcribing the resulting length-controllable poly(A) tail.^149^

In mammalian cells, actively translated mRNAs generally contain 100–250 adenosine residues.^117,150^ A poly(A) tail of optimal length can improve translation efficiency and mRNA stability.^150,151^ Studies have shown that when poly(A) tail size increases to 120 bp, the expression level of the corresponding protein increases accordingly. However, when poly(A) tail size is greater than 120 bp, the expression level of the corresponding protein did not increase.^152^ In addition, other design strategies of poly(A) tails exist. For instance, BioNTech uses a segmented poly(A) tail whose two-tail structures are connected in tandem by a 10 bp UGC linker sequence (A30LA70).^27,153^ Current studies have shown that the segmented poly(A) tail extends mRNA half-life and improves translation efficiency compared to the long-chain poly(A) tail.^154^

#### Modified nucleosides

Naturally occurring modified nucleosides are found in mRNAs in humans.^155^ The host immune system can easily recognize unmodified mRNA or by-products formed during IVT of engineered mRNA as exogenous molecules.^74^ dsRNA can activate PRR such as TLR3,^52,156^ whereas ssRNA activates TLR7 and TLR8 to produce IFN-I, thus inducing inflammation in the host and interrupting mRNA translation.^157,158^ To avoid this, Karikó and colleagues found that adding modified nucleosides during IVT of mRNA can significantly reduce the host inflammatory response without affecting protein expression.^81,159^ Currently, the following modified nucleosides are available for mRNA modification: pseudouridine (ψ), N^1^-methylpseudouridine (m^1^ψ), 5-methoxyuridine (mo^5^U), 2-thiouridine (s^2^U), 5-methylcytidine (m^5^C) and N6-methyladenosine (m^6^A) (Fig. 4).^115,160–170^ Studies have found that replacing original nucleosides with m^6^A and s^2^U inhibits activation of TLR3, whereas activation of TLR7 and TLR8 is blocked when using ψ, 5-methyluridine (m^5^U), m^6^A, and s^2^U, thereby inhibiting the innate immune responses and improving protein translation efficiency.^81,159,171^ Kormann and colleagues^152^ replaced 25% of mRNA cytosine with m^5^C and 25% of uridine with s^2^U, which improved mRNA stability and increased protein translation in mice. However, replacing natural nucleosides in the right proportion might be challenging, which might hinder vaccine quality control and consistency. Currently, nucleoside-modified mRNA vaccines employ 100% chemically modified nucleosides replacing natural nucleosides, and m^1^ψ is often used to replace uridine during IVT to improve the safety and stability of mRNA vaccines.^52,134,172^

At present, mRNA vaccines that have entered clinical trials or been approved for commercialization include both nucleoside-modified and nucleoside-unmodified mRNA with sequence-optimized mRNA. Nucleoside-modified mRNA can reduce the activation of TLRs, retinoic acid-inducible gene I (RIG-I), protein kinase R (PKR), and 2′-5′-oligoadenylate synthetase (OAS). Additionally, nucleoside modification increases translation activity and resistance against RNase L-mediated degradation.^54,81,173–176^ Many nucleoside-modified mRNA COVID-19 vaccines, including BNT162b2, BNT162b1, and BNT162b3 developed by BioNTech^27,177^; mRNA-1273, TAK919, mRNA-1273.211, mRNA-1273.351, mRNA-1283 developed by Moderna^28,178^; ChulaCov19 developed by Chulalongkorn University; and PTX-COVID19-B developed by Providence Therapeutics, have been approved for commercialization or entered clinical trials.^104^ In addition, nucleoside-modified mRNA vaccines have been widely used in vaccine development for other viral agents such as Cytomegalovirus (CMV), Respiratory syncytial virus (RSV), Influenza A virus, Chikungunya virus, Zika virus, Dengue virus.^92,98,179–182^

In contrast, studies on mRNA vaccines with unmodified nucleosides have yielded inconsistent conclusions. Thess and colleagues showed that unmodified, GC-rich mRNA engineered with an optimized UTR sequence yielded more sustained antigen expression compared to nucleoside-modified mRNA.^183^ In contrast, Pardi and colleagues reported that protein levels after intradermal injection of m^1^ψ-modified mRNA in mice were 20 times higher compared to sequence-optimized but unmodified mRNA.^184^ Among unmodified-nucleoside COVID-19 vaccines currently in clinical trials are included CVnCoV developed by CureVac (clinical trial terminated), ARCoV developed by Abogen,^185^ BNT162a1 developed by BioNTech,^130^ and MRT5500 developed by Translate Bio (clinical trial terminated).^104^ A vaccine platform (RNActive®) was designed by CureVac^186^ combining the use of co-delivered RNA and protamine (a polycationic peptide) complex as adjuvant, which has been shown to effectively trigger innate immune responses and enhance vaccine immunogenicity.^186–189^ Using this technique, the Rabies vaccine CV7201 was developed by CureVac and is currently in phase I clinical trial.^190^

In summary, chemical modifications regulate the functional specificity of biological macromolecules, and to date, a total of 16 modifications have been found in eukaryotic mRNA. Both Moderna and BioNTech use pseudouridine modifications to ensure mRNA stability in their COVID-19 vaccine formulation.

### saRNA

Engineered saRNA vaccines rely on the insertion of the gene encoding the target antigen into the genome of an RNA virus (mainly alphavirus) as well as the use of its replication machinery to amplify the delivered RNA, thereby increasing antigen expression.^191–193^ In terms of structures, in addition to the conventional elements of non-replicating mRNA, saRNA contains a long ORF after 5′ UTR encoding the four NSPs (nsP1, nsP2, nsP3, and nsP4) of alphavirus that functions as an mRNA capping enzyme, an NTPase/helicase/protease, a macrodomain, or an RNA-dependent RNA polymerase (RDRP), respectively. A subgenomic promoter can then be used to initiate transcription of the gene coding for the target antigen.^194^ Once in the cytoplasm of a host cell, saRNA undergoes translation by the endogenous ribosomal machinery, thereby enabling translation of nsP precursors to form an early replication complex. The positive-strand RNA is then used as a template to synthesize negative-strand RNA, which is the replication intermediate. With the cleavage of nsP precursors, a late replication complex is produced. Then, the negative-strand RNA of the replication intermediate is used as a template to synthesize a full-length positive-strand genomic RNA. At the same time, a subgenomic positive-strand RNA containing only information coding for the antigen is also synthesized (Fig. 3). As a result, one copy of saRNA produces multiple copies of RNA transcripts by the above-described mechanism to initiate self-amplification of antigen genes in the cell.^35,195,196^

The idea of using in vitro synthesized saRNA as a preventive vaccine was first proposed by Zhou and colleagues in 1994, using a modified Semliki Forest virus (SFV) replicon to express the nucleoprotein (NP) of the influenza virus.^197^ Subsequently, Fleeton and colleagues used the same SFV replicon to develop saRNA vaccines for influenza A virus, RSV, and louping ill virus (LIV). After direct intramuscular injection in mice, the naked saRNA could induce protective immune response.^198^ Decades later, Geall and colleagues were the first to use LNP to encapsulate a saRNA chimera composed of the Venezuelan equine encephalitis virus (VEEV) and Sindbis virus (SINV) replicons into a vaccine which was used to immunize mice, and immunogenicity was significantly improved compared with unencapsulated group.^199^ In recent years, several viral replicons have been used in saRNA vaccines, such as those of VEEV, classical swine fever virus (CSFV), tick-borne encephalitis virus (TBEV).^96,200–203^ In previous studies, the alphavirus genome have been screened and multiple superior mutations that could improve and optimize RNA replicons have been identified.^204–209^ Li and colleagues developed an in vitro evolution strategy, and six mutations (namely A1979G, G3936C, A4311G, A4758G, G4796T, G4944A) were identified in the nsP2 and nsP3 of the VEEV replicon, which were shown to promote expression of subgenomic RNA in cells.^210^ Moreover, saRNAs have yielded promising results in preclinical research for COVID-19 vaccines. Recently, two different saRNA vaccines developed independently by Arcturus Therapeutics and Imperial College London have shown favorable immune responses against SARS-CoV-2, and have entered clinical trials.^211–213^

The most advantageous aspect of saRNA vaccine is that it can be produced with ultra-low doses of saRNA. Compared with the dose of mRNA in the Moderna vaccine (100 μg) and that of Pfizer-BioNTech vaccine (30 μg), the amount of saRNA required for vaccine development is within a range of 0.1~10 μg.^212^ This ultra-low injection dose has several advantages^214^: (i) greater production potential, since the same amount of raw materials and the same equipment yield more vaccine production; (ii) reduced side effects considering the lower dosage; (iii) allows combination with other vaccines due to its lower dosage; (iv) intrinsic adjuvant effect; (v) high levels of antigen expression and long-term duration of immunity. saRNA vaccines have nonetheless certain shortcomings, including the risk of excessive activation of the inflammatory response, and the production of viral nsP produced by the alphavirus replicon that may interfere with normal signal transduction in target host cells.^214,215^ In addition, considering that the length of nsP1–4 sequence is approximately 7 kb, the full length of a saRNA sequence is usually above 9 kb, which might hinder cloning construction. Hence, the delivery vector employed in saRNA vaccines must allow for higher loading capacity and encapsulation efficiency.^35^

### taRNA

taRNA is a self-amplified RNA composed of two separate RNA molecules (Fig. 3). To circumvent the problems caused by large and complex sequences of saRNA, the R&D team of the Imperial College London has developed a split replicon (splitzicon) system which enables encoding the alphavirus nsPs and the heterologous gene of interest (GOI) on separate RNA molecules whilst conserving the self-amplification properties of the replicon RNA.^216^ Blakney and colleagues^216^ used fluorescent reporter genes as encoding proteins and designed positive and negative splitzicons to identify structural components affecting self-amplification characteristics of VEEV replicons, thus providing a new strategy for developing saRNA vaccines based on alphavirus RNA replicons. In a recent study, Beissert and colleagues developed a novel bipartite vector system using taRNA,^111^ containing a transreplicon expressing hemagglutinin antigen (TR-HA) of influenza virus obtained by deleting the replicase gene in the amplified RNA of alphavirus together with an optimized non-replicating mRNA carrying a replicase gene. After application of the resulting vaccine in mice, it was shown that 0.05 μg of taRNA resulted in complete protection comparable to non-replicating mRNA vaccine or saRNA vaccine.

taRNAs usually yield safer vaccines compared to saRNA vaccines. The alphavirus replicon gene is divided into two different RNA molecules encoding vaccine antigens, which reduces the possibility of transfer of recombinant virus particles to host cells. In addition, taRNA technology has potential advantages in transfer capacity, versatility, and production scale-up, thus showing promising applications.^217^ At present, taRNA technology has only been applied in preclinical studies for influenza vaccines.^111^ COVID-19 vaccines based on taRNA technology have not been reported.

### CircRNA

CircRNA is a highly stable single-stranded RNA with a covalently closed loop structure (Fig. 3), including a large category of non-coding RNAs generated by backsplicing in eukaryotic cells.^218–220^ In the 1970s, Sanger and colleagues discovered single-stranded circRNA viruses in higher plants.^221^ Later, circRNA was also identified in yeast mitochondria and hepatitis D virus.^222^ Despite the lack of essential elements for cap-dependent translation, circRNA can be translated by adding the IRES element or m^6^A modification incorporated to its 5′ UTR region.^223,224^ Unlike linear RNA, circRNA offers several advantages in vaccine development. The covalently closed loop structure of circRNA protects from exonuclease degradation, thus increasing circRNA half-life and stability. Moreover, previous studies have reported that cell transfection efficiency was maintained when circRNA was kept at room temperature for two weeks.^112^ In addition, unmodified circRNA has been shown to induce TLR/RIG-I-mediated innate immune response compared to unmodified linear mRNA.^225,226^

Recently, Liang and colleagues^112^ developed a circRNA vaccine against SARS-CoV-2 encoding a trimeric receptor-binding domain (RBD) of the spike protein of SARS-CoV-2, considering that in RBD the signal peptide sequence of human tissue plasminogen activator (tPA) was fused to the N-terminus to ensure antigen secretion, whereas the trimerization motif of bacteriophage T4 fibritin protein (foldon) was fused to the C-terminus to ensure the native conformation of the antigen protein trimer. In addition, the IRES element was inserted before the coding gene to initiate translation, and circRNA was produced using a group I ribozyme. Finally, LNP was used for encapsulation to obtain a circRNA vaccine. After immunizing mice with the obtained vaccine, long-lasting neutralizing antibodies and Th1-biased cellular immune responses were produced. Moreover, the vaccine also showed neutralizing activity against the Beta variant (B.1.351). Liang and colleagues^227^ further improved the circRNA vaccine by constructing multiple circRNAs based on several SARS-CoV-2 variants; the results revealed that circRNA prepared with sequences of Delta strains resulted in broad-spectrum protection and production of neutralizing antibodies against both Delta and Omicron. However, vaccines produced with circRNA prepared based on Omicron sequences provided a narrower protection, and produced neutralizing antibodies could protect only against Omicron but not against Delta. In addition, vaccination with circRNA prepared with the original SARS-CoV-2 strain sequence followed by a booster dose of the vaccine containing circRNA prepared with Delta sequences conferred good protection against Delta and Omicron. Since RBD is the main region inducing the production of neutralizing antibodies, it can be speculated that the future development of COVID-19 vaccines should focus on the Delta variant.

In summary, although considered a byproduct of the mRNA splicing process, circRNA has now emerged as an important new class of non-coding RNAs. With its highly stable properties without nucleotide modification, circRNA can potentially become a novel platform for vaccine and drug development.

## Antigen design for COVID-19 mRNA vaccines

**Fig. 6:**
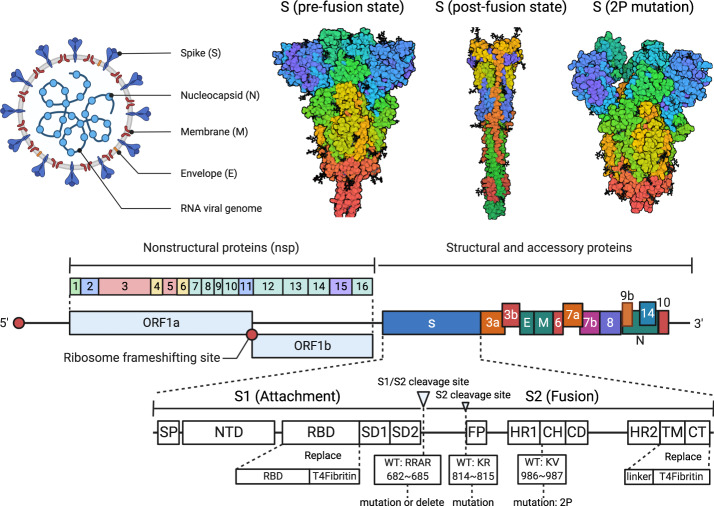
Rationale underlying the design strategy of COVID-19 mRNA vaccine. Representation of the SARS-CoV-2 reference genome showing structural, nonstructural, and accessory proteins, consisting of ORF1a, ORF1b, Spike protein (S), ORF3a, ORF3b, Envelope (E), Membrane (M), ORF6, ORF7a, ORF7b, ORF8, ORF9b, ORF14, Nucleocapsid (N) and ORF10.^485^ Spike and receptor-binding domain (RBD) proteins are mainly used as target antigens for the design and optimization of COVID-19 mRNA vaccines. This figure is created with BioRender.com

The trimeric S protein on the surface of SARS-CoV-2 plays a key role in mediating host cell invasion. Therefore, the S protein is considered the main antigen for vaccine design.^228–230^ The S protein is cleaved into S1 and S2 subunits during viral infection by the Furin enzyme and transmembrane serine protease 2 (TMPRSS2).^231,232^ The S1 subunit comprises the signal peptide (SP), RBD, N-terminal domain (NTD), C-terminal domain 1 (CTD1), and C-terminal domain 2 (CTD2), and primarily interacts with the cellular angiotensin-converting enzyme 2 (ACE2) receptor via RBD. The S2 subunit comprises the fusion peptide (FP), double heptad repeat (HR), central helix region (CH), connector domain (CD), transmembrane domain (TMD), and cytoplasmic tail (CT), and the S2 subunit is responsible for mediating the fusion between the virus and the host cell membrane^233–236^ (Fig. 6). Upon binding of RBD and the ACE2 receptor on the host cell membrane, the S protein undergoes a structural rearrangement that results in a postfusion conformation.^236^ Studies have found that the S protein prefusion conformation induces better immunogenicity and can be therefore considered an ideal target antigen.^237^ Most neutralizing antibodies are molded based on epitopes of S protein in prefusion conformation, which is covered once the S protein acquires the postfusion conformation, thus reducing the production of neutralizing antibodies.^238^

The ORF containing the coding sequence that is translated into protein in vivo is the most critical component of the mRNA vaccine. To improve the safety, efficacy, and stability of mRNA vaccines, researchers usually performed codon optimization^172,239–243^ on the antigen-coding sequence to enhance translation efficiency. Optimization of mRNA secondary structure^244,245^ and stability can be achieved by increasing the GC content of the coding sequence.^246–248^ Codon preference varies extensively in different organisms,^249^ therefore adjusting the balance between codon usage frequency and host tRNA availability can significantly improve translation efficiency and in vivo expression of the target antigen.^250,251^ Several online codon optimization tools are available,^252–266^ and optimization algorithms have been conceived for different research purposes.^267^ A few studies have indicated that optimization algorithms designed by BioNTech and Moderna may have shortcomings. In addition, since mRNA vaccines are often injected intramuscularly, a better immune response can be expected if codon optimization is adjusted for skeletal muscle preference.^153^

At present, two strategies are commonly used for designing COVID-19 S protein: 2P mutation and S1/S2 Cleavage site (Fig. 6). The 2 P mutation strategy is based on the findings of studies exploring the S protein in prefusion conformation in Middle East Respiratory Syndrome Coronavirus (MERS-CoV),^268^ SARS-CoV,^269^, and human coronavirus HKU1 (HCoV-HKU1).^270^ By adopting this strategy, two amino acids at the top of the helical position of the S2 subunit center are substituted with prolines (K986P and V987P), which was shown to improve stability of S protein in prefusion conformation effectively. The 2P mutation method is applicable to SARS-CoV-2^271^ and other β-coronavirus viruses,^28^ and BioNTech, Moderna, CureVac, and other developers have all adopted the 2P mutation strategy.^27,28,185,272^ The S1/S2 cleavage site strategy employs direct deletion of the sequence Q677TNSPRRARYSV687 in wild-type SARS-CoV-2 protein S to Q677TILRYSV683^238^ or mutation of amino acids (RRAR to GGSG)^141^ that ultimately prevent the S protein from cleavage in the host cell, thus maintaining its structural stability and inducing stronger immune responses. In addition to RiboBio mutation of the S1/S2 cleavage site (682–685: RRAR to GGSG),^141^ antigen design for the protein found in the COVID-19 recombinant vaccine developed by Novavax also introduced similar mutations (682-685: RRAR to QQAQ) to maintain the stability of S protein conformation.^16,273–275^

Certain research institutions have developed unique strategies for optimizing the S protein (Fig. 6), such as deleting TMD, CT, FP on the S2 subunit and mutating the S2′ cleavage site (K814A, R815N) to improve the conformational stability of prefusion S protein.^141^ In certain cases, an additional sequence can be inserted in the anterior segment of the ORF region to increase the expression of antigenic proteins.^276^ Moreover, since the S protein is trimeric, studies have shown that the trimeric motif of T4 bacteriophage fibritin introduced at the 3′ end of the coding region of the S protein or RBD protein can mimic the native structure of S protein and enhance antigen immunogenicity.^141,277,278^ Furthermore, previous studies revealed significant differences in protein expression levels of target antigens when different signal peptides are selected.^93^ In the COVID-19 recombinant vaccine developed by WESTVAC, expression of RBD protein was enhanced when the GP67 signal peptide was used.^15^ The S protein signal peptide MFVFLVLLPLVSSQCV has been used in COVID-19 mRNA vaccines by several developers, including BioNTech and Moderna. However, other signal peptides have also been used. For example, RiboBio employs the immunoglobulin heavy chain variable region (IGVH) signal peptide sequence (MDWIWRILFLVGAATGAHS) in COVID-19 mRNA vaccines to increase target protein expression. In brief, since mRNA vaccines involve sequence editing, structure, stability, and expression of the S protein may be modified to improve spatial conformation and thus vaccine-induced immune response (Table 2).

**Table 2:** Antigen design strategies adopted for COVID-19 mRNA vaccines

Developers/Vaccine Name	Antigen	Nucleotide modification	2Pmut	S1/S2 Cleavage site	Additional design	Reference(s)
BioNTech/BNT162b2	Spike	+	+	−	NA	^27^
Moderna/mRNA1273	Spike	+	+	−	NA	^272^
CureVac/CVnCoV	Spike	−	+	−	RNActive® technology	^480^
RiboBio	Spike	+	+	+	T4 Fibritin; S2 mut; Delete FP, TMD, CTD	^141^
Abogen/ARCoV	RBD	+	NI		NA	^185^
BioNTech/BNT162b1	RBD	+	NI		T4 Fibritin	^278^
CanSinoBIO	RBD	+	NI		RBD-CTB fusion protein; RBD-CRM197 fusion protein; CPG adjuvant; TLR adjuvant	^481^
Stemirna	Spike; S1 subunit; RBD; M; N; E	+	−	−	Insert additional sequences before ORF; LPP delivery systems	^276,330^
LIVERNA	Spike; S1 subunit; RBD	+	NA	NA	NA	^479^
Institute of Microbiology, Chinese Academy of Sciences	Spike; S1 subunit; RBD	+	NA	NA	NA	^482^

2P mut: two proline mutations (K986P, V987P) on the S2 subunit of the S protein to maintain its stability; *NA*: not applicable; *NI*: not involved; *CTB*: cholera toxin B subunit; *CPG*: non-methylated short nucleotides cytosine and guanine; *TLR*: toll-like receptor; *FP*: fusion peptide; *TMD*: transmembrane domain; *CTD*: C-terminal domain; *RBD*: receptor binding domain; *LPP*: lipopolyplex.

## Delivery systems

Passage of mRNA through the phospholipid bilayer of the cell membrane is difficult due to its large molecular weight (10^4^–10^6^ Da), negative charge, and proneness to degradation by nucleases. Therefore, in recent years, various delivery vehicles have been developed for mRNA encapsulation, including LNPs, polyplexes and polymeric nanoparticles, lipopolyplexes (LPPs), and cationic polypeptides. Lipids and their derivatives are considered a new delivery system for mRNA vaccines and have been attracting much attention due to their low immunogenicity, biocompatibility, and high encapsulation rate. As an early version of LNPs first discovered in 1965,^279^ liposomes are the earliest nanomedicine delivery platform to pass from concept to clinical application successfully.^280^ The next generation of LNPs, which includes solid LNPs, nanostructured lipid carriers, and cationic lipid-nucleic acid complexes,^281–283^ possesses more complex internal structures, stability, and targeting capacity. In addition to vaccines, these substances can be used as a new drug delivery platform for anticancer and nucleic acid therapeutics.

### LNPs

**Fig. 7:**
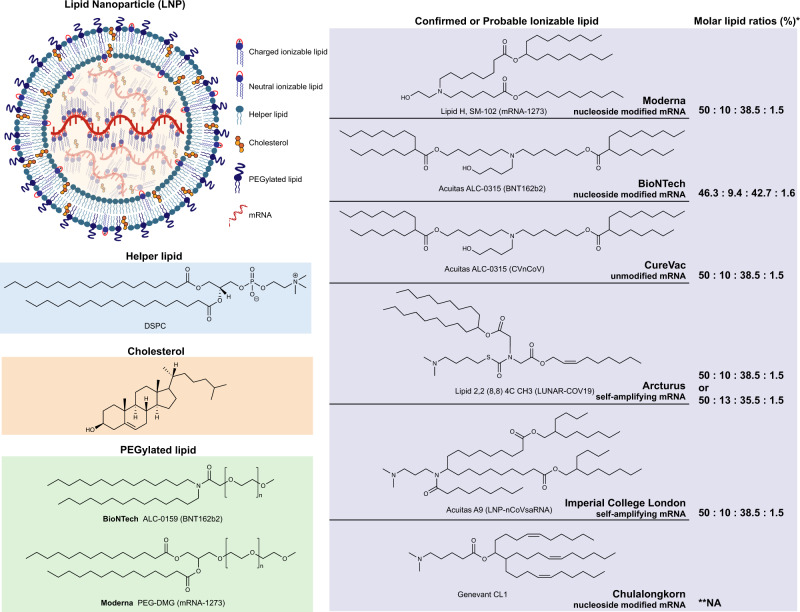
Structure of lipid nanoparticles (LNPs) and lipid components employed in currently available COVID-19 mRNA vaccines. LNPs are composed of four components: ionizable lipid, helper lipid, cholesterol, and PEGylated lipid. Binding with mRNA occurs by the ionizable lipid that occupies the central core of the LNP. PEGylated lipid is found on the surface of LNPs along with helper lipid forming the bilayer. Cholesterol, charged ionizable lipids, and neutral ionizable lipids are distributed throughout LNPs. The confirmed or the most likely chemical structure of ionizable lipids employed in COVID-19 mRNA vaccines developed by Moderna, BioNTech, CureVac, Arcturus, Imperial College London, and Chulalongkorn University.^289^ *Molar lipid ratio (%) of ionizable lipid: helper lipid: cholesterol: PEGylated lipid; **NA: Not applicable

LNPs is a nano-scale vesicle which simulates the lipid structure of the cell membrane and can encapsulate mRNA in its cavity, being considered the most investigated mRNA vaccine delivery system. Currently, most COVID-19 mRNA vaccine candidates use LNPs as the delivery system. LNPs are composed of four components: ionizable lipids, helper phospholipids, cholesterol, and PEGylated lipids, among which, ionizable lipids are considered the key components. COVID-19 mRNA vaccines designed by different developers vary widely in structural design (Fig. 7).

The cationic lipid N-[1-(2,3-dioleyloxy)propyl]-N,N,N-trimethylammonium chloride (DOTMA) was first used by Malone and colleagues to transfect mRNA into cells.^75^ Although DOTMA has high delivery efficiency in vitro, it is quickly cleared in the host blood circulation and has pro-inflammatory and pro-apoptotic toxic effects.^284^ Accordingly, since ionizable lipids are positively charged in an acidic buffer environment, they can bind to negatively charged RNA and facilitate endosomal escape of mRNA after entering the host cell. Ionizable lipids are neutral at physiological pH, thus making them safer and more stable for use as delivery systems in vaccines.^285^ DLin-MC3-DMA is the ionizable lipid used in LNP formulation in Onpattro®^286^ the first-ever siRNA drug approved by the FDA. Moderna used DLin-MC3-DMA ionizable lipids to prepare mRNA vaccines for Zika virus and influenza on which preclinical and clinical studies were conducted.^90,287,288^ However, it was later found that the di-linoleic alkyl tail in DLin-MC3-DMA is prone to degradation, and repeated booster doses can potentially lead to cumulative toxicity.^289^ Based on these observations, Moderna has developed an ionizable lipid (namely Lipid H, SM-102), whose tail adopts larger branches which in turn increases potency, and whose introduced ester bonds increase biodegradability (Fig. 7).^290,291^ In contrast, BioNTech uses ALC-0315, whose chemical structure is similar to SM-102, as the ionizable lipid in the LNP formulation of their COVID-19 mRNA vaccine. (Fig. 7).^289,292^

Although ionizable lipids are essential components of LNPs, the other three components (i.e., helper phospholipids, cholesterol, and PEGylated lipids) play an important role in LNPs assembly and function. Helper phospholipids are amphiphilic lipids that support the lipid bilayer structure, help promote fusion with endosomal membranes, and determine the specificity of target organs.^293,294^ The choice of helper phospholipid for LNPs formulation is highly dependent on the length of the delivered RNA molecule. For instance, saturated helper lipids (such as 1,2-Dioctadecanoyl-sn-glycero-3-phophocholine —DSPC) are indicated for the transport of short siRNAs,^295^ whereas unsaturated lipids (e.g., 1,2-dioleoyl-sn-glycero-3-phosphoethanolamine —DOPE) are more conducive to delivering longer mRNAs.^296,297^ However, DSPC is employed as helper phospholipid in the formulation of both Moderna and BioNTech COVID-19 mRNA vaccines (Fig. 7),^27,298–300^ which may be justified by the fact that DSPC performs better than DOPE when combined with ionizable lipids, as well as that DSPC is the only FDA-approved molecule for LNP formulation.^104^

As a naturally occurring lipid, cholesterol can modulate the bilayer structure of biological membranes in various ways by altering fluidity, thickness, compressibility, water penetration capacity, and intrinsic curvature.^283,301^ In LNPs formulations, cholesterol increases stability by filling gaps among LNPs molecules and aiding fusion with endosomal membranes, thereby promoting uptake of the vaccine complex.^302^ A previous study showed that LNPs made from oxidatively modified cholesterol can deliver mRNA to the liver microenvironment in a targeted manner.^303^

The PEGylated lipid is the least abundant component in LNPs formulation and is composed of hydrophilic polyethylene glycol (PEG) and a hydrophobic anchoring lipid [dimyristoyl Phosphoethanolamine (DMPE) or dimyristoyl glycerol (DMG)]. Both substances affect the size, permeability, and immunogenicity properties of LNPs. The main role of PEGylated lipid in LNPs is to reduce aggregation and non-specific uptake by immune cells.^304–306^ The molecular weight of PEG is typically 350–3000 Da,^41^ and the tail length of the anchoring lipid is typically 10−18 carbon,^307^ which are parameters that determine how extensively LNPs will circulate in the host as well as their uptake rate by immune cells. The greater the tail length of the anchoring lipid, the longer the half-life of the LNP complex in the host, and the lower the probability of being assimilated by macrophages in a non-specific manner.^104^ PEG2000-DMG is used in LNPs formulation of the FDA-approved siRNA drug Onpattro^TM^ and Moderna COVID-19 mRNA vaccine (Fig. 7).^308^

### Other delivery systems

Polymers are another widely used mRNA delivery system, and offer better physical stability than lipid carriers. Three main types of polymer-based delivery vehicles have been described previously: poly(ethylene imine) (PEI), poly(L-lysine) (PLL), and poly (amidoamine) (PAMAM), among which only PEI has been widely used as a delivery system in mRNA vaccines.^96,309–314^ Although the optimized chemical structure of PEI has higher gene transfection efficiency, it still induces strong cytotoxicity due to the high cationic charge density. Targeted modification of PEG chains can reduce cytotoxicity and significantly improve delivery efficiency in vivo and in vitro.^315–317^

In addition, another challenge with using polymer delivery vehicles is their biodegradability,^318^ which required the use of polyesters as mRNA carriers,^316,319–321^ among which are included poly(beta-amino esters) (PBAE), poly(amine-co-esters) (PACE), and poly(lactic acid) (PLA). Su and colleagues^322^ employed encapsulation of PBAE with lipids in which mRNA loaded on the surface of LNPs by electrostatic interaction. PBAE biocompatibility conferred by lipids facilitates cell entry. In addition, the pH-responsive and biodegradability of PBAE facilitate endosomal escape of delivered mRNAs and minimize cytotoxicity. Recently, Blakney and colleagues^323^ obtained a disulfide-linked poly(amidoamine) polymer (named pABOL), which can be used to generate polydisperse nanocomplexes of 100 nm diameter in size. In addition, in vivo experiments in mice showed that the delivery efficiency of pABOL is higher than that of PEI carriers. The pABOL system, which was developed by the Imperial College London R&D team, is considered a delivery system with the saRNA of SARS-CoV-2 in the vaccine formulation. However, its delivery efficiency was 1,000 times lower than that of LNP employed in the Acuitas vaccine formulation.^289,324^ Although certain characteristics of polymers, such as relatively low delivery efficiency and innate heterogeneity, limit its clinical application and industrial production, it has potential application prospects and areas for improvement.

Lipid shell-coated LPPs are a ternary complex containing a condensed mRNA core packaged in a lipid shell.^325,326^ LPPs have higher stability, low cytotoxicity, cell delivery and endosomal escape efficiency.^327–329^ Shen and colleagues^326^ developed a PbAE-based LPP platform that can efficiently deliver mRNA; in this delivery system, the PbAE-mRNA complex is encapsulated in a lipid shell which is mainly composed of 1,2dioleoyl-sn-glycero-3-ethylphosphocholine (EDOPC)/DOPE/1,2distearoyl-sn-glycero-3-phosphoethanolamine-N-[amino(polyethylene glycol)-2000] (DSPE-PEG2k). The findings of this study revealed that, compared with naked PbAE-mRNA, cellular transfection efficiency was greatly improved when LPP-coated PbAE-mRNA was used. Moreover, this LPP-based mRNA vaccine exhibited intrinsic adjuvant activity, which stimulates dendritic cells (DCs) to secrete cytokines and inhibit tumor growth by activating the TLR7/8 signaling pathway, resulting in a significant antitumor activity. Yang and colleagues^330^ applied similar LPP technology to encapsulate mRNA in two steps using ionizable lipid, DOPE and PEG-lipid to generate COVID-19 mRNA vaccine with a core-shell structure, which showed significant protection in mice and non-human primates.

In addition to lipid and polymer carriers, peptides can also be used for mRNA delivery.^331–333^ Since some amino acids carry cationic or amphiphilic amino groups, they can electrostatically bind mRNA to form nanocomplexes. A commercial peptide, PepFect14, was shown to effectively deliver therapeutic mRNAs to ovarian tumor cells in mice.^334^ In addition, protamine was shown to activate TLR7 and TLR8 pathways, thereby showing potential as a delivery vehicle with adjuvant effect for vaccines or gene therapy.^188^ Based on this observation, the protamine-containing delivery platform developed by CureVac has been used in various vaccines and gene therapy for cancer treatment.^85,335^ Finally, cationic squalene emulsions can be applicable for mRNA delivery.^336,337^ These nanoemulsions are composed of a squalene-based core and a lipid shell. Squalene has an adjuvant effect, and cationic lipids on the surface of the lipid shell can bind to mRNA by electrostatic adsorption. The Lipid InOrganic Nanoparticles (LION) delivery vehicle developed by HDT Bio is composed of squalene, Span 60, Tween80, cationic lipid 1,2-dioleoyl-3-trimethylammonium propane (DOTAP), and superparamagnetic iron oxide (SPIO), and has been used to deliver self-replicating mRNA encoding the S protein of SARS-CoV-2. Preclinical results indicate that this delivery vehicle can improve vaccine stability, delivery efficiency and immunogenicity, thereby inducing strong neutralizing antibodies and T cell response in mice and non-human primates.^338^

## Progress in clinical research on mRNA Vaccines

With the advent of mRNA delivery systems and nucleic acid modification technology, research on mRNA technology for cancer treatment as well as prevention of several infectious diseases has progressed rapidly. mRNA vaccines have shown good efficacy in the treatment of acute myelocytic leukemia (AML),^339–341^ non-small cell lung cancer (NSCLC),^342,343^ and melanoma^344–348^ (Table 3). The mRNA vaccine BNT111 developed by BioNTech’s FixVac platform to treat advanced melanoma has entered phase II clinical trials and was assigned the FDA Fast Track designation on November 19, 2021.^349^ The mRNA contained in the BNT111 vaccine encodes the four tumor-associated antigens (TAAs)—NY-ESO-1, MAGE-A3, tyrosinase, and TPTE—delivered in an RNA-lipoplex formulation. Previous studies have demonstrated that the use of BNT111 alone or in combination with PD-1 antibody can activate tumor antigen-specific CD4^+^ and CD8^+^ T cells and elicit durable immune responses.^345,350–352^ Additionally, the mRNA cancer vaccine CV9201 developed by CureVac encoding five NSCLC antigens has entered phase I/IIa clinical trials comprising 7 patients with locally advanced NSCLC and 39 patients with metastatic NSCLC. Specific immune responses against at least one antigen were detected in 63% of patients after treatment, and the frequency of activated IgD^+^ CD38^hi^ B cells increased by more than two-fold in 60% of evaluated patients.^343,353,354^ Moderna’s mRNA personalized cancer vaccine mRNA-4157 is comprised of 34 unique neoantigen genes (encoded by tumor-specific mutated genes) combined in a single mRNA vaccine; this vaccine was proven safe and tolerable in combination with pembrolizumab in phase I clinical trials. Finally, the overall response rate (ORR) for the treatment of 10 cases of HPV-negative head and neck squamous cell carcinoma (HPV^-^HNSCC) with the mRNA-4157 vaccine was 50%, of which 2 cases achieved complete remission (CR).^355–358^

**Table 3:** mRNA vaccine candidates for cancer therapy currently in clinical trials

Sponsor	Cancer type	Identifier	Drug administration	Phase	Status
Duke University	Glioblastoma, malignant glioma	NCT00626483	CMV pp65-LAMP mRNA-loaded DC + GM-CSF	I	Completed
		NCT00639639	CMV-ALT + CMV pp65-LAMP mRNA-loaded DC	I	Active, not recruiting
		NCT02529072	DC loaded with CMV Ag mRNA in combination with nivolumab	I	Completed
		NCT02366728	Human CMV pp65-LAMP mRNA-pulsed autologous DCs	II	Active, not recruiting
	Glioblastoma	NCT00890032	BTSC mRNA-loaded DCs	I	Completed
		NCT03927222	Human CMV pp65-LAMP mRNA-pulsed autologous DCs + temozolomide + Td toxoid + GM-CSF	II	Suspended
		NCT03688178	Human CMV pp65-LAMP mRNA-pulsed autologous DCs + temozolomide + varlilumab + Td toxoid + 111In-labeled DCs + unpulsed DCs	II	Recruiting
	Melanoma	NCT01216436	DCs transfected with mRNA encoding TAAs	I	Terminated
Radboud University	Melanoma	NCT00929019	Autologous DCs EP with mRNA encoding gp100 and tyrosinase	I/II	Terminated
		NCT00243529	Autologous DCs transfected with mRNA encoding TAAs	I/II	Completed
		NCT00940004	DCs EP with mRNA encoding TAAs gp100 and tyrosinase	I/II	Completed
		NCT01530698	Autologous DCs EP with mRNA	I/II	Completed
		NCT02285413	DCs loaded with mRNA encoding TAAs gp100 and tyrosinase +/− cisplatinum	II	Completed
	Colorectal cancer	NCT00228189	CEA mRNA-loaded DCs	I	Completed
	Hematological Malignancies	NCT02528682	MiHA mRNA-loaded PD-L-silenced DC	I/II	Completed
	Prostatic Neoplasms	NCT02692976	DCs loaded with protamine/mRNA encoding KLH + DCs loading with MHC I binding peptides, NY-ESO-1 and MUC1 PepTivator	II	Completed
Oslo University Hospital	Melanoma	NCT00961844	DCs - transfected with hTERT-, survivin- and tumor cell derived RNA + ex vivo T cell expansion and reinfusion+temozolomide	I/II	Terminated
		NCT01278940	mRNA-transfected DCs + IL-2	I/II	Completed
	Prostate cancer	NCT01197625	Autologous DCs loaded with mRNA from primary prostate cancer tissue, hTERT, and survivin	I/II	Active, not recruiting
		NCT01278914	mRNA-transfected DCs	I/II	Completed
	Glioblastoma	NCT00846456	Tumor stem cell-derived mRNA-transfected DCs	I/II	Completed
		NCT03548571	DCs transfected with mRNA encoding survivin and hTERT + temozolomide	II/III	Recruiting
	Ovarian cancer	NCT01334047	DCs loaded with amplified ovarian cancer stem cell mRNA, hTERT, and survivin	I/II	Terminated
Antwerp University Hospital	AML	NCT00834002	WT1mRNA-transfected autologous DCs	I	Completed
		NCT01686334	DCs EP with autologous WT1 mRNA	II	Recruiting
	AML, CML, multiple myeloma	NCT00965224	DCs EP with autologous WT1 mRNA	II	Unknown
	Multiple solid tumors	NCT01291420	WT1 mRNA-EP autologous DCs	I/II	Unknown
	Mesothelioma	NCT02649829	DCs loaded with WT1 + chemotherapy	I/II	Recruiting
	Glioblastoma	NCT02649582	Autologous WT1 mRNA-loaded DCs + temozolomide	I/II	Recruiting
Argos Therapeutics	Renal cell carcinoma	NCT01482949	DC EP with autologous tumor mRNA +/− sunitinib	II	Terminated
		NCT00678119	DCs co-EP with CD40L IVT RNA and autologous total tumor RNA + sunitinib	II	Completed
		NCT00272649	DCs co-EP with CD40L IVT RNA and autologous total tumor RNA	I/II	Completed
		NCT01582672	DCs EP with Autologous tumor mRNA plus sunitinib	III	Terminated
		NCT00087984	Autologous tumor total RNA-transfected DCs	I/II	Completed
	Pancreatic cancer	NCT00664482	Autologous DCs EP with tumor total RNA	NA	Completed
BioNTech	Melanoma	NCT01684241	Naked RNA encoding TAAs	I	Completed
		NCT02035956	Personalized poly-epitopic RNA-based vaccine	I	Completed
		NCT02410733	Lipo-MERIT, encoding for 4 melanoma associated non-mutated antigens	I	Active, not recruiting
		NCT04526899	RNA-LPX with NY-ESO-1, MAGE-A3, tyrosinase, and TPTE +/− cemiplimab	II	Recruiting
	Breast cancer	NCT02316457	RNA-LPX with TNBC TAAs, p53, and neo-Ags	I	Active, not recruiting
	Prostate cancer	NCT04382898	RNA-LPX with prostate TAAs +/− cemiplimab	I/II	Recruiting
CureVac	Prostate cancer	NCT02140138	CV9104 with or without needle-free injection device	II	Terminated
		NCT00831467	RNActive TAAs mRNA CV9103	I/II	Completed
		NCT01817738	RNActive TAAs mRNA CV9104	II/II	Terminated
	NSCLC	NCT00923312	RNActive TAAs mRNA CV9201	I/II	Completed
		NCT01915524	RNActive TAAs mRNA CV9202 + local radiation	I	Terminated
Guangdong 999 Brain Hospital	Glioblastoma	NCT02808364	Autologous DCloaded with TAA mRNA	I/II	Unknown
		NCT02709616	Autologous DC loaded with TAA mRNA	I/II	Unknown
	Brain cancer	NCT02808416	Personalized cellular vaccine	I	Unknown
Herlev Hospital	Breast cancer, melanoma	NCT00978913	DCs transfected with hTERT, survivin, and p53	I	Completed
	Prostate cancer	NCT01446731	DCs transfected with PSA, PAP, survivin, and hTERT mRNA+docetaxel	II	Completed
Life Research Technologies	Ovarian cancer	NCT01456065	DCs loaded with TERT-mRNA and survivin-peptide	I	Unknown
Ludwig-Maximilian-University of Munich	AML	NCT01734304	DCs EP with mRNA encoding WT1, PRAME, and CMVpp65	I/II	Completed
MD Anderson Cancer center	AML	NCT00514189	Autologous DCs loaded with AML lysate and mRNA	I	Terminated
Memorial Sloan Kettering Cancer Center	Melanoma	NCT01456104	Autologous LCs EP with mRNA encoding TAA	I	Active, notrecruiting
	Multiple myeloma	NCT01995708	CT7, MAGE-A3, and WT1 mRNA-EP LCs	I	Active, notrecruiting
Universitair Ziekenhuis Brussel	Melanoma	NCT01066390	DCs EP with TAA and TriMix mRNA	I	Completed
		NCT01302496	DCs EP with TAA and TriMix mRNA + ipilimumab	II	Completed
		NCT01676779	DC EP with TAA and TriMix mRNA	II	Completed
University Hospital Erlangen	Melanoma	NCT01983748	Autologous DCs loaded with tumor mRNA	III	Recruiting
University Hospital Tübingen	Melanoma	NCT00204516	mRNA encoding autologous melanoma associated antigens+GM-CSF	I/II	Completed
		NCT00204607	mRNA encoding MART-1, tyrosinase, gp100, MAGEA1, MAGE-A3 and survivin+GM-CSF	I/II	Completed
	Recurrent prostate cancer	NCT02452307	Peptide vaccine + montanide ISA-51+/−GM-CSF+/− imiquimod +/− mRNA/protamin	I/II	Unknown
University of Campinas	AML, myelodysplastic syndromes	NCT03083054	Autologous DCs EP with WT1 mRNA	I/II	Active, not recruiting
University of Florida	Prostate cancer	NCT00906243	CV9103 encoding 4 prostate specific antigens	I/II	Terminated
	Glioblastoma, Malignant Glioma	NCT02465268	pp65-shLAMP mRNA DCs + GM-CSF	II	Recruiting
	Metastatic Prostate Cancer	NCT01153113	hTERT mRNA transfected DCs	I/II	Withdrawn
Ludwig Institute for Cancer Research	Metastatic NSCLC	NCT03164772	RNActive TAAs mRNA CV9202 + durvalumab +/−tremelimumab	I/II	Completed
Stemirna Therapeutics	Esophageal Cancer, NSCLC	NCT03908671	Personalized mRNA vaccine encoding neoAg	NA	Not yet recruiting
Hospital Affiliated to the Academy of Military Medical Sciences	Esophagus Cancer	NCT02693236	Adenovirus-transfected autologous DCs + CIK cells	I/II	Unknown
	NSCLC with bone metastases	NCT02688686	SOCS1, MUC1 and survivin mRNA-loaded DCs + cytokine-induced killer	I/II	Unknown
University Medical Center Groningen	Ovarian Cancer	NCT04163094	RNA-LPX with ovarian TAAs + carboplatin/paclitaxel	I	Recruiting
ModernaTX, Inc.	Melanoma	NCT03897881	mRNA-4157 encoding neoAg + pembrolizumab	II	Recruiting
	Solid tumors	NCT03313778	mRNA-4157 encoding neoAg +/− pembrolizumab	I	Recruiting
Asterias Biotherapeutics	AML	NCT00510133	DCs transfected with hTERT mRNA with a LAMP-1 targeting sequence	II	Completed
National Cancer Institute	Melanoma, Colon Cancer, Gastrointestinal Cancer, Genitourinary Cancer, Hepatocellular Cancer	NCT03480152	Personalized cancer mRNA vaccine NCI-4650	I/II	Terminated
Changhai Hospital	Esophageal Squamous Carcinoma, Gastric Adenocarcinoma, Pancreatic Adenocarcinoma, Colorectal Adenocarcinoma	NCT03468244	Personalized mRNA vaccine encoding neoAg	NA	Recruiting

*AML*: acute myeloid leukemia; *WT1*: Wilms tumor 1; *CML*: chronic myeloid leukemia; *DCs*: dendritic cells; *EP*: electroporated; *CD40L*: CD40 ligand; *IVT*: in vitro transcribed; *hTERT*: human telomerase reverse transcriptase; *LAMP-1*: lysosome-associated membrane protein 1; *TNBC*: triple-negative breast cancer; *TAA*: tumor-associated antigen; *CMV*: cytomegalovirus; *GM-CSF*: granulocyte-macrophage colony-stimulating factor; *BTSC*: brain tumor stem cell; *Td*: tetanus-diphtheria; *PSA*: prostate-specific antigen; *PAP*: prostatic acid phosphatase; *PRAME*: melanoma antigen preferentially expressed in tumors; *LCs*: langerhans cells; *CEA*: carcinoembryonic antigen; *KLH*: keyhole limpet hemocyanin; TriMix: CD40L, CD70, and constitutively active TLR4 mRNA; *NA*: not applicable; SOCS: suppressor of cytokine signaling; *neoAg*: personalized neoantigen; *NSCLC*: non-small-cell lung cancer.

In addition to mRNA cancer vaccines, studies on mRNA vaccines to prevent infectious diseases have gradually expanded. Several mRNA vaccine candidates for viral agents other than SARS-CoV-2 have entered clinical trials (Table 4), including CMV,^359^ influenza virus,^288,360^ rabies virus,^86,88^ Zika virus,^90,361–364^ RSV,^365,366^ human metapneumovirus (hMPV).^367^ Currently, five mRNA vaccines for influenza virus encoding the HA antigen have entered clinical trials: mRNA-1851, mRNA-1440, and mRNA-1010^368,369^ from Moderna; MRT-5400 and MRT-5401 co-developed by Sanofi and Translate Bio.^370^ Current clinical trials showed that the mRNA-1440 vaccine against H10N8 and the mRNA-1851 vaccine against H7N9 influenza viruses were well tolerated and elicited robust humoral immune responses when tested separately (Table 4).^360^

**Table 4:** mRNA vaccine candidates for infectious diseases currently in clinical trials

Sponsor(s)/Name	Virus type (Administration route)	Antigen type	Phase	Identifier	Status
ModernaTX, Inc./mRNA-1647	CMV (i.m)	CMV pentamer and glycoprotein B	III	NCT05085366	Recruiting
			II	NCT04975893	Enrolling by invitation
			II	NCT04232280	Active, not recruiting
			I	NCT05105048	Not yet recruiting
			I	NCT03382405	Completed
ModernaTX, Inc./mRNA-1443	CMV (i.m)	CMV-associated	I	NCT03382405	Completed
Massachusetts General Hospital, NIAID/Undefined	HIV (DC loaded, i.d)	HIV-associated	I/II	NCT00833781	Completed
Fundacion Clinic per a la Recerca Biomédica/iHIVARNA-01	HIV (DC loaded; i.nod)	HIV-associated with TriMix	II	NCT02888756	Terminated
	HIV (NA)		I	NCT02413645	Completed
Argos Therapeutics/AGS-004	HIV (DC EP; i.d)	HIV-associated Ag and CD40L	II	NCT00672191	Completed
			II	NCT01069809	Completed
			I/II	NCT00381212	Completed
			I	NCT02042248	Completed
			I	NCT02707900	Terminated
ModernaTX, Inc./mRNA-1893	Zika virus (i.m)	PrM-E	II	NCT04917861	Recruiting
			I	NCT04064905	Completed
ModernaTX, Inc./mRNA-1325	Zika virus (i.m)	PrM-E	I	NCT03014089	Completed
ModernaTX, Inc./mRNA-1010	Influenza A virus (H1N1 and H3N2 subtypes), Influenza B virus (Yamagata lineage, Victoria lineage) (i.m)	NA	I/II	NCT04956575	Recruiting
ModernaTX, Inc./mRNA-1851(VAL-339851)	Influenza A virus (H7N9 subtype) (i.m)	H7N9 HA	I	NCT03345043	Completed
ModernaTX, Inc./mRNA-1440 (VAL-506440)	Influenza A virus (H10N8 subtype) (i.m)	H10N8 HA	I	NCT03076385	Completed
Translate Bio, Sanofi/MRT-5400	Influenza A virus (H3N2 subtype) (i.m)	H3N2 HA	I	Unregistered	Unregistered
Translate Bio, Sanofi/MRT-5401	Influenza A virus (H3N2 subtype) (i.m)	H3N2 HA	I	Unregistered	Unregistered
CureVac/ CV7201	Rabies virus (i.d, i.m)	Rabies G protein	I	NCT02241135	Completed
CureVac/ CV7202	Rabies virus (i.m)	Rabies G protein	I	NCT03713086	Active, not recruiting
GSK/ GSK3903133A	Rabies virus (i.m)	Rabies G protein	I	NCT04062669	Active, not recruiting
ModernaTX, Inc./mRNA-1345	RSV (i.m)	Stabilized prefusion F glycoprotein	I	NCT04528719	Recruiting
ModernaTX, Inc./mRNA-1777(V171)	RSV (i.m)	Stabilized prefusion F glycoprotein	I	Unregistered	Unregistered
ModernaTX, Inc./mRNA-1172(V172)	RSV (i.m)	Stabilized prefusion F glycoprotein	I	Unregistered	Unregistered
ModernaTX, Inc./mRNA-1944	Chikungunya virus (i.m)	Chikungunya mAb	I	NCT03829384	Completed
ModernaTX, Inc./mRNA-1388(VAL-181388)	Chikungunya virus (i.m)	NA	I	NCT03325075	Completed
ModernaTX, Inc./mRNA-1653	hMPV (i.m)	Fusion proteins of hMPV and PIV3	I	NCT04144348	Recruiting
			I	NCT03392389	Completed

*CMV*: cytomegalovirus; *HIV*: human immunodeficiency virus; *NIAID*: National Institute of Allergy and Infectious Diseases; *DCs*: dendritic cells; *NA*: not applicable; *EP*: electroporated; *HA*: hemagglutinin; *GSK*: GlaxoSmithKline; *RSV*: Respiratory syncytial virus; *mAb*: monoclonal Antibody; *hMPV*: human metapneumovirus; *PIV3*: parainfluenza virus type 3; *i.m*: intramuscular; *i.d*, intradermal; *i.nod*, intranodal.

In 2021, a rare and highly contagious SARS-CoV-2 variant emerged. SARS-CoV-2 was first identified in late 2019 and has constantly been evolving, as multiple new variants have emerged since then. To facilitate monitoring and investigation, WHO has divided the SARS-CoV-2 variants into three classes: variants of concern (VOCs), variants of interest (VOIs), and variants under monitoring (VUMs). For VOCs, 4 variants, Alpha (B.1.1.7), Beta (B.1.351), Gamma (P.1), and Delta (B.1.617.2), are included.^371^ Each variant determined the rise of a new wave of COVID-19 infections, resulting in a massive spike in the number of deaths worldwide. On November 26, 2021, a new variant termed Omicron (B.1.1.529) was designated the fifth VOC by the WHO, immediately triggering a global health alert.^372–374^ To limit the spread of the current pandemic, governments from different countries have launched a special review task and approval of new drugs into clinical trials which showed promising application in COVID-19 vaccine production.^375^

BioNTech and Pfizer collaborated to develop five COVID-19 mRNA vaccine candidates at the beginning of the pandemic, which were based on nucleoside-modified mRNA (BNT162b2, BNT162b1, BNT162b3), non-modified mRNA (BNT162a1) and self-amplifying mRNA (BNT162c2). Till now, all of these candidates have entered clinical trials (Table 5).^130,376,377^ Among them, three different antigen types were designed in the nucleoside modified mRNA: transmembrane prefusion spike (BNT162b2), secreted spike RBD (BNT162b1), transmembrane spike RBD (BNT162b3), among which the first two were considered lead candidates; BNT162b2 encodes a full-length spike glycoprotein with two proline mutations in the S2 subunit, which is designed to maintain the protein in pre-fusion conformation; BNT162b1 encodes a secreted form of the trimeric spike protein RBD. In a phase I clinical trial, 195 subjects were divided into two groups based on the administered vaccine (BNT162b1 or BNT162b2); each group was further divided based on vaccination doses: individuals in the BNT162b1 group received either two doses with 10, 20, or 30 μg of the immunogen, or a single dose with 100 μg, or the placebo; individuals in the BNT162b2 group received either two doses with 10, 20, or 30 μg of the immunogen, or the placebo. Clinical trial data indicated that both mRNA vaccine candidates induced neutralizing antibodies at a comparable level. However, the vaccine developers eventually decide to proceed with BNT162b2 to phase II/III international clinical trials in view of its milder reactogenicity.^277,278,378^ BioNTech/Pfizer proceeded with BNT162b2 in global phase II/III clinical trials on July 27, 2020, which received EUA by the US government on December 11, 2020^379,380^ as well as the European Union conditional marketing authorization (CMA) on December 21, 2020.^381,382^ The BNT162b2 mRNA vaccine was then added to the WHO Emergency Use Listing (EUL) on December 31, 2020,^383^ and approved on May 10 and May 28, 2021 by the US government and the European Union, respectively, for administration in adolescents aged 12 to 15.^384^ The US FDA officially authorized BNT162b2 for vaccination of individuals aged 16 and older on August 23, 2021.^105^ The FDA granted BNT162b2 emergency approval for use in children and adolescents aged 5 to 11 on October 29, 2021.^103,106^

**Table 5:** mRNA vaccine candidates for COVID-19 currently in clinical trials

Vacine name/Developer(s)	Antigen/Delivery vehicles	Route of administration/Schedule/Dose	Phase	Identifier (Number of participants; Location)	Outcomes
**mRNA type: nucleoside-modified**					
BNT162b2/BioNTech,Pfizer	Transmembrane prefusion spike/LNP	IM/Day 0 + 21/30 μg	IV	NCT05057182 (300 participants; Hong Kong)	Fully approved for use in individuals aged 16 or older^483^; EUA for use in individuals aged 5 or older^106^; EUA for use as a single booster in individuals aged 18 or older^395,396^; 95% overall efficacy^385^
				NCT04852861 (150 participants; Belgium)	
				NCT04952766 (240 participants; France)	
				NCT04961229 (504 participants; Not Provided)	
				NCT05057169 (400 participants; Hong Kong)	
				NCT04969250 (640 participants; Nigeria, Spain, Switzerland, Uganda, United States)	
				NCT05168709 (60 participants; Australia)	
				NCT04775069 (900 participants; Hong Kong)	
			III	NCT04816669 (610 participants; USA)	
				NCT04805125 (431 participants; Switzerland)	
				NCT04800133 (900 participants; Hong Kong)	
				NCT04713553 (1,530 participants; USA)	
			II/III	NCT04368728 (43,998 participants; Argentina, Brazil, Germany, South Africa, Turkey, USA)	
				NCT04754594 (700 participants; Brazil, South Africa, Spain, UK, USA)	
			II	ISRCTN73765130 (2,886 participants; UK)	
				NCT04894435 (1,200 participants; Canada)	
				NCT04761822 (3,400 participants; USA)	
				NCT04824638 (300 participants; France)	
				NCT04860739 (676 participants; Spain)	
				EUCTR2021-001978-37 (600 participants; Spain)	
				NCT04649021 (950 participants; China)	
				ISRCTN69254139 (820 participants; UK)	
				NCT04907331 (3,000 participants; Austria)	
				NCT04895982 (360 participants; Brazil, Germany, USA)	
			I/II	EUCTR2020-001038-36, NCT04380701 (476 participants; Germany)	
				NCT04889209 (800 participants; USA)	
				NCT04588480 (160 participants; Japan)	
			II	NCT04839315 (100 participants; USA)	
				NCT04816643 (4, 500 participants; Finland, Poland, Spain, USA)	
mRNA-1273/Moderna, NIAID, BARDA	Transmembrane prefusion spike/LNP	IM/Day 0 + 28/100 μg	IV	NCT04952402 (700 participants; Puerto Rico, United States)	EUA obtained in several countries; EUA as a single booster in individuals aged 18 or older^396^; 100% efficacy against B.1.1.7; 95.7% efficacy against B.1.35 in Qatar^417^; 94.1% efficacy at preventing COVID-19 infections, including severe cases in the US^410^
				NCT04969250 (640 participants; Nigeria, Spain, Switzerland, Uganda, United States)	
				NCT05030974 (460 participants; Netherlands)	
				NCT05079633 (220 participants; Taiwan)	
				NCT04978038 (414 participants; Canada, Ontario)	
				NCT04760132 (10,000 participants; Denmark)	
			III	NCT04811664 (37,500 participants; USA)	
				NCT04470427 (30,420 participants; USA)	
				NCT04860297 (240 participants; USA)	
				NCT04806113 (220 participants; Canada)	
				NCT04805125 (431 participants; Switzerland)	
			II/III	NCT04649151 (3,732 participants; USA)	
				NCT04796896 (6,975 participants; USA)	
			II	ISRCTN73765130 (2,886 participants; UK)	
				NCT04847050 (120 participants; USA)	
				NCT04894435 (1,200 participants; Canada)	
				NCT04748471 (180 participants; France)	
				NCT04761822 (3,400 participants; USA)	
				NCT04405076 (660 participants; USA)	
			I/II	NCT04889209 (800 participants; USA)	
			I	NCT04785144 (135 participants; USA)	
				NCT04813796 (125 participants; USA)	
				NCT04839315 (100 participants; USA)	
				NCT04283461 (120 participants; USA)	
BNT162b1/BioNTech,Pfizer	Secreted spike RBD/LNP	IM/Day 0 + 21/10, 20, 30 or 100 μg	II/III	NCT04368728 (43,998 participants; Argentina, Brazil, Germany, South Africa, Turkey, USA)	8–50-fold increase in GMCs of RBD-binding IgG; 1.9–4.6-fold neutralizing GMTs compared to the convalescent panel; higher rate of systemic events compared to BNT162b2^278^
			II/II	EudraCT 2020-001038-36, NCT04380701 (476 participants; Germany)	
			I	ChiCTR2000034825, NCT04523571(144 participants; China)	
BNT162b3/BioNTech,Pfizer	Transmembrane spike RBD/LNP	IM/Day 0 + 21/30 μg	I/II	NCT04537949, EUCTR2020-003267-26- DE (96 participants; Germany)	Unknown
mRNA-1273.211/Moderna, NIAID, BARDA	Transmembrane prefusion spike/LNP	IM/Day0 + 28/20, 50 μg	II	NCT04405076 (660 participants; USA)	Increased neutralizing GMTs when used as a booster^415^
mRNA-1273.351/Moderna, NIAID, BARDA	Transmembrane prefusion spike/LNP	IM/Day0 + 28/20 or 50 μg	I	NCT04785144 (135 participants; USA)	Increased neutralizing GMTs when used as a booster^415^
mRNA-1283/Moderna, NIAID, BARDA	Transmembrane prefusion spike/LNP	IM/Day0 + 28/NA	I	NCT04813796 (125 participants; USA)	Unknown
TAK-919/Takeda, Moderna	Transmembrane prefusion spike/LNP	IM/Day0 + Day29/100 μg	I/II	NCT04677660 (200 participants; Japan)	Approved in Japan^484^
ChulaCov19/Chulalongkorn University	Transmembrane spike/LNP	IM/Day0 + 21/10, 25 or 50 μg	I/II	NCT04566276 (96 participants; Thailand)	Unknown
PTX-COVID19-B/Providence Therapeutics	Transmembrane spike/LNP	IM/Day0 + 28/16, 40 or 100 μg	I	NCT04765436 (60 participants; Canada)	High neutralization titers against VOCs^432^
**mRNA type: unmodified nucleosides**					
CVnCoV/CureVac	Transmembrane prefusion spike/LNP	IM/Day0 + 29/12 μg	III	NCT04652102, EUCTR2020-003998-22(39,693 participants; Argentina, Belgium, Colombia, Dominican Republic, Germany, Mexico, Netherlands, Panama, Peru, Spain)	48.2% efficacy^425^; EMA terminated rolling review^429^
				NCT04860258 (1,200 participants; Belgium)	
				NCT04848467 (1,000 participants; Argentina, Colombia, Peru)	
			II	ISRCTN73765130 (2,886 participants; UK)	
				NCT04515147, PER-054-20 (674 participants; Panama, Peru)	
			I	NCT04449276 (280 participants; Belgium, Germany)	
ARCoV/Abogen, Walvax Biotechnology, PLA	Secreted spike RBD/LNP	IM/ Day0 + 28/15 μg	III	NCT04847102 (28,000 participants; China, Mexico)	2-fold neutralizing GMTs compared to convalescent panel.^439^
			II	ChiCTR2100041855(420 participants; China)	
			I	ChiCTR2000034112(568 participants; China)	
BNT162a1/BioNTech, Pfizer	Secreted spike RBD/LNP	IM/NA/NA	I/II	EudraCT 2020-001038-36, NCT04380701 (476 participants; Germany)	Unknown
MRT5500/Sanofi, Translate Bio	Transmembrane prefusion spike/LNP	IM/Day0 + 21/NA	I/II	NCT04798027 (333 participants; Honduras, USA)	Terminated^436^; 91–100% seroconversion rate^437^
**mRNA-type: self-amplifying RNA**					
ARCT-021/Arcturus Therapeutics	Transmembrane prefusion spike/LNP	IM/Day0 + 28/5.0 μg or 7.5 μg	II	NCT04668339 (600 participants; Singapore, USA)	Seroconversion in most participants^441^
				NCT04728347 (106 participants; Singapore)	
			I/II	NCT04480957 (92 participants; Singapore)	
ARCT-165/Arcturus Therapeutics	NA/LNP	IM/Day0 + 29/ NA	I/II	NCT05037097 (72 participants; Singapore, USA)	Unknown
ARCT-154/Arcturus Therapeutics	NA	IM/Day0 + 29/ 5 μg	I/II/III	NCT05012943 (2,1000 participants; Vietnam)	Unknown
BNT162c2/BioNTech, Pfizer	Transmembrane prefusion spike/LNP	IM/Day0 + 21/ NA	I/II	EudraCT 2020-001038-36, NCT04380701 (476 participants; Germany)	Unknown
LNP-nCoV saRNA/Imperial College London, Acuitas Therapeutics	Transmembrane prefusion spike/LNP	IM/NA/0.1~10 μg	I	ISRCTN17072692 (320 participants; UK)	39–61% seroconversion rate^212^
EXG-5003/lixirgen Therapeutics/ Fujita Health University	NA/LNP	ID/Day0/NA	I/II	NCT04863131 (60 participants; Japan)	Unknown
HDT-301/SENAI CIMATEC; HDT	NA/LION	IM/Day0 + 28/ 1 μg, 5 μg or 25 μg	I	NCT04844268 (90 participants; NA)	Unknown
LNP-nCOV saRNA02/RC/ UVRI and LSHTM Uganda Research Unit	NA/LNP	IM/Day 0 + 28/ 5.0 μg	I	NCT04934111 (42 participants; Uganda)	Unknown
SAM-LNP-S/Gristone Oncology, NIAID	Transmembrane spike/LNP	IM/Day0 + 30 or Day0 + 85~130/ 30 μg or 3 μg	I	NCT04776317 (147 participants; USA)	Unknown
CoV2 SAM (LNP)/GlaxoSmithKline	Transmembrane spike/LNP	IM/Day0 + 30/ 1.0 μg	I	NCT04758962 (10 participants; USA)	Unknown

*IM*: intramuscular; *ID*: intradermal; *BARDA*: Biomedical Advanced Research and Development Authority; *EUA*: emergency use authorization; *LNP*: lipid nanoparticle; *NIAID*: National Institute of Allergy and Infectious Diseases; *PLA*: People Liberation Army; *RBD*: receptor-binding domain; *VOCs*: variant of concerns; *GMCs*: geometric mean concentrations, *GMTs*: geometric mean titers; *NA*: not applicable; Clinical trials are regularly updated, therefore locations and the number of participants of clinical trials reported above are subjected to change.

BNT162b2 is administered intramuscularly as a two-dose scheme of 30 μg of immunogen per dose, 21 days apart. The efficacy of the vaccine at preventing COVID-19 infection in individuals aged 16 was 95%, and over the six subsequent months, vaccine efficacy was 91.3%.^385–387^ Studies have shown that the protective effect of BNT162b2 against SARS-CoV-2 infection peaks after the second dose and then quickly declines, with the humoral immune response sharply decreasing.^388–391^ However, results from clinical trials with over 5,000 people who had received BioNTech/Pfizer booster injections showed that protection conferred by the vaccine was 95.6%.^392–394^ On September 22, 2021, the FDA approved the BNT162b2 vaccine for administration in high-risk populations as a booster,^395^ then expanded eligibility on November 19, 2021 as a single booster of both BNT162b2 and mRNA-1273 to individuals aged 18 and older after completion of the primary vaccination scheme with any FDA-authorized or previously approved COVID-19 vaccine.^396^ In a mass vaccination study which included 3,159,136 participants from Israel, vaccine effectiveness of BNT162b2 as a two-dose scheme was 94%.^397^ However, the vaccine was associated with increased risk of myocarditis in Israeli participants, reaching a rate of approximately 3 events per 100,000 persons.^398–400^ Another study indicated that the incidence of anaphylaxis after BNT162b2 vaccination in Japan was higher, which points towards considering race-related adverse effects^401^ whose underlying causes are still unknown. The PEG additive,^402^ which is also used in several cosmetics and pharmaceutical drugs, has been incriminated as a possible cause for anaphylaxis induced by the BNT162b2 mRNA vaccine. This is related to the fact 57% of the 37 people who presented anaphylaxis had a history of allergy, and four had a history of cosmetic allergy, suggesting a potential role for PEG in inducing anaphylaxis.

The SARS-CoV-2 variant B.1.617.2 was first identified in India in December 2020, being later designated the Delta variant, and became predominant in several countries.^403^ Real-world data from Qatar indicated that the BNT162b2 vaccine had only 51.9% effectiveness against the Delta variant, which was significantly lower compared to 75.0% and 89.5% effectiveness conferred by the vaccine against Beta and Alpha variants, respectively.^102^ As mentioned previously, protection conferred by BNT162b2 decreases significantly over time. The effectiveness of the BNT162b2 vaccine against the Beta variant was measured shortly after the population of Qatar had been vaccinated, whereas the effectiveness of BNT162b2 against the Delta variant was conducted several months after the second dose, which could be one of the reasons for low effectiveness of BNT162b2 against the Delta variant.^404,405^ More recently, and using a pseudovirus neutralization test (pVNT), neutralization titers induced after two doses of BNT162b2 were 160, 7, 24, and 73 GMTs for wild-type SARS-CoV-2, Omicron, Beta, and Delta variants, respectively, but improved to 368, 164, 279, and 413 GMTs, after one month following a booster vaccination. Comparable trends were observed in live virus neutralization testing. Thus vaccine booster with BNT162b2 may enhance neutralization of the Omicron variant.^406^ Similar findings suggest that vaccination with three doses of the mRNA vaccine BNT162b2 may protect against Omicron-mediated COVID-19.^407,408^

The mRNA-1273 developed by Moderna encodes the full-length prefusion spike protein of SARS-CoV-2 and is the second mRNA vaccine received EUA by the US government on December 18, 2020, and received Biologics License Application (BLA) on Janurary 31, 2022.^409^ Results of clinical trials have shown that mRNA-1273 is generally well tolerated and safe to use in adolescents and adults. No serious safety concerns have been identified so far, and most adverse events were mild or moderate; the most common adverse effect was pain at the vaccine injection site on both shots, whereas headache, fatigue, myalgia and chills were adverted after the second shot.^410,411^ In a phase III clinical trial involving 30,420 people, participants aged 18 or older were given two doses of 100 μg of mRNA-1273 with a 28-day interval, and vaccine efficacy was 94.1% (Table 5), with similar immune responses among adolescents aged 12 to 17.^410,412–414^ The mRNA-1273.351 developed based on SARS-CoV-2 Beta variant first identified in South Africa, together with the mRNA-1273.211 molecule containing both mRNA-1273.351 and mRNA-1273 has entered phase III clinical trials as a vaccine booster. Approximately six months after administration of the two injections of the mRNA-1273 vaccine, each group of twenty participants received a booster with the immunogen: 50 μg mRNA-1273, mRNA-1273.211 or mRNA-1273.351. The neutralization effect of the booster in each immunization group against SARS-CoV-2 variants Beta, Gamma and Delta reached a level comparable to that observed against the wild-type D614G strain. Among the three booster vaccines evaluated, the multivalent mRNA-1273.211 induced the largest geometric mean titers (GMT) for variants Beta, Gamma and Delta.^415,416^ Between December 28, 2020, and May 10, 2021, 256,037 people in Qatar received at least one dose of the mRNA-1273 vaccine, whereas 181,034 people received two doses. Real-world data showed that effectiveness of mRNA-1273 against Alpha and Beta variants was 100% and 96.4%, respectively,^417,418^ whereas effectiveness against Delta was slightly lower (73.1%).^102^ Other studies have shown that mRNA-1273 is still quite effective in congregate settings or higher-risk exposure such as prisons or hospitals in which the Delta variant was prevalent.^419,420^ Further real-world data on vaccine effectiveness for Omicron and Delta variants showed that administration of three doses of mRNA-1273 provided a high and durable protection against Delta infection (95.2%) but lower protection against Omicron (62.5%). However, none of the vaccinated individuals with three doses of mRNA-1273 were hospitalized, which indicates a promising alternative.^421^ Overall, despite the fact that vaccine effectiveness against SARS-CoV-2 decreases over time, vaccination with BNT162b2 and mRNA-1273 was still effective in preventing infection with Delta and other variants, reducing hospitalization and mortality, with mRNA-1273 showing a slightly superior performance compared to BNT162b2.^420,422–424^

Additionally, CureVac’s first-generation COVID-19 mRNA vaccine candidate (CVnCoV) is administered as a two-dose series with 12 μg, 28 days apart. Preliminary results from phase IIb/III clinical trials showed that the overall efficacy of the vaccine was 48.2% (Table 5), which failed to meet prespecified success criteria.^425^ In previously preclinical trials, CVnCoV was shown to induce high levels of neutralizing antibodies in rodents and non-human primates, outbalancing immune responses mediated by CD4^+^ and CD8^+^ T cells as well as showing better efficacy against SARS-CoV-2 D614G variant.^426^ Phase I clinical data showed that CVnCoV has acceptable tolerance and high immunogenicity.^29,427^ The low performance of CVnCoV in phase II clinical trials has been attributed to the rise of multiple SARS-CoV-2 variants. In phase II clinical trials, 228 COVID-19 cases were reported, from which 204 have been sampled for whole-genome sequencing, which showed that only 3% of the patients had been infected by wild-type SARS-CoV-2, whereas 14 variants have been identified in the remaining patients.^425^ The low vaccination dosage of CVnCoV and the use of unmodified nucleotides might have contributed to the performance of this vaccine in phase II clinical trials compared to the licensed COVID-19 vaccine.^428^ On October 12, 2021, the European Medicines Agency (EMA) announced the suspension of the rolling review of CVnCoV.^429^ On the same day, CureVac announced that they would abandon CVnCoV follow-up clinical studies to focus their research efforts on a second-generation mRNA vaccine candidate developed in collaboration with GSK.^430^

The COVID-19 vaccine PTX-COVID19-B produced by Providence Therapeutics in Canada has entered phase II clinical trial, proposing a two-dose scheme with 40 μg per injection, 28 days apart. PTX-COVID19-B was shown to induce high titers of neutralizing antibodies against wild-type SARS-CoV-2 and variants, including Alpha, Beta, and Delta, which were comparable to those elicited by the approved COVID-19 mRNA vaccines when assessed by the same neutralization assay.^431,432^ Moreover, Sanofi Pasteur collaborated with Translate Bio to develop the first-generation vaccine MRT5500, which encodes the full-length spike protein of SARS-CoV-2. Results of preclinical trials showed that MRT5500 induced a Th1-biased immune response in mice and non-human primates and prevented Th2-bias response which can induce vaccine-related enhanced respiratory disease (VAERD).^433–435^ Mid-term results of phase I clinical trial of MRT5500 showed that the seroconversion rate was 91% to 100%. However, considering that global supply of COVID-19 vaccines is sufficient, Sanofi decided to abandon MRT5500 follow-up studies.^436,437^ In addition, the mRNA vaccine ARCoV developed in China by Abogen has entered phase III clinical trials^438^; ARCoV is reportedly safe and well-tolerated at an amount of 15 μg, which induced high titers of neutralizing antibodies titers (approximately two-fold higher that those of patients which had recovered from COVID-19 infection).^439^ Moreover, mRNA vaccines of Stemirna and LIVERNA have also entered phase I and II clinical trials.^440^

Furthermore, the saRNA vaccine LNP-nCoVsaRNA developed by the Imperial College London showed seroconversion rate from 8% to 61% as determined by ELISA when 0.1–10.0 μg was administered per dose group, demonstrated inferior immunogenicity in humans compared to that observed in mice.^212^ In addition, the results of phase I/II clinical trials of the saRNA vaccine ARCT-021 developed by Arcturus Therapeutics indicated the production of robust anti-spike specific antibodies when 5.0–7.5 μg was administered per dose group, but it also failed to reach 100% seroconversion rate.^441,442^ saRNA vaccines have dosage advantages compared to the approved mRNA vaccines BNT162b2, mRNA-1273, and other non-replicating mRNA vaccines. However, current results of clinical trials suggest that immunogenicity profiles of saRNA vaccines may not be comparable to those obtained with non-self-replicating mRNA vaccines. This might likely reflect differences in exogenous RNA restriction by the innate immune sensing. Thus, incorporation of encoded modulators of human PRR or of a wider range of potential modifications may positively affect immunogenicity and efficacy of saRNA vaccines.^443,444^

## Production and quality control of mRNA vaccines

### Production of mRNA vaccines

**Fig. 8:**
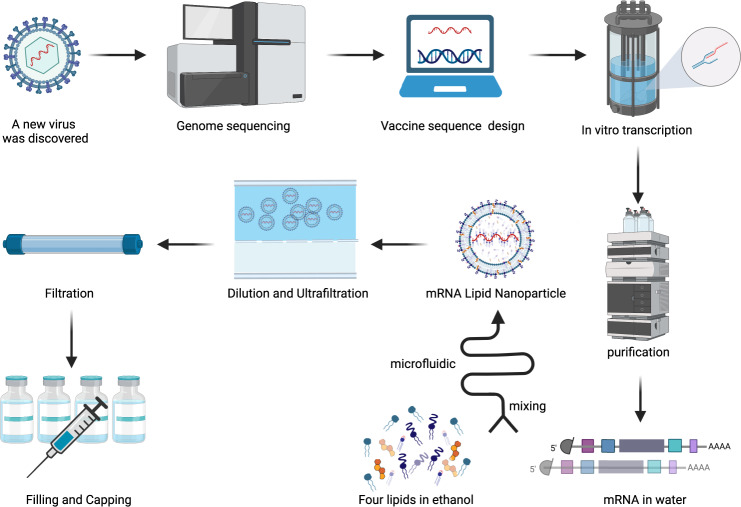
Production process of mRNA vaccines. The design of an mRNA vaccine is conditioned to the definition of the antigen sequence of the target pathogen. By determining the target antigen and optimizing its coding sequence, the mRNA can be transcribed in vitro by RNA polymerase. The synthesized mRNA is purified by different processes and then mixed with a lipid phase using microfluidics and encapsulated into an mRNA-lipid nanoparticle (mRNA-LNP) complex. Subsequently, self-assembly of LNPs is completed by dilution and concentration by ultrafiltration. Finally, after sterile filtration, filling, and capping, the mRNA vaccine is obtained

Production of mRNA vaccines does not require culturing cells or viruses as in traditional vaccine production technology, relying instead on in vitro synthesis technology. Therefore, the production cycle is shorter and easy to scale up, hence offering the possibility of quick industrialization of vaccine production. From IVT of mRNA to preparation of mRNA-LNP complexes, the entire production cycle for an mRNA vaccine might last approximately 10 days. Considering also the time required for qualification and release, the product can be available on the market within 40 days. As a technology platform, mRNA vaccine technology is broadly compatible with any mRNA sequences and virtually all vaccines based on proteins can be produced using this technology. The production process mainly involves the following steps: target antigen sequence design; plasmid construction; establishing a three-level bacterial biobank; DNA template preparation; IVT of mRNA; mRNA purification; LNP formulation and encapsulation; mRNA-LNP complex dilution; mRNA-LNP complex concentration; sterile filtration and filling; and other minor final steps (Fig. 8).^445^ The production of mRNA vaccines is carried out under conditions that comply with current laws, regulations, and management guidelines preconized by governments and regulatory authorities in various countries. In this context, the five main elements (man, material, machine, method, and environment) must meet local and international requirements of good manufacturing practices and other standards.^446–450^

The production of mRNA vaccines starts with the synthesis of the target antigen. After the antigen gene sequence is optimized and cloned into a plasmid, engineered bacteria are amplified and cultured to retrieve the desired gene sequences. Two strategies can be employed to obtain the linearized DNA template for IVT: using restriction enzymes to linearize the plasmid, or using PCR to amplify target DNA in preclinical studies or small-scale production. The purified and recovered DNA template is then used in IVT to obtain the corresponding mRNA. Using linear DNA as a template, NTPs are employed as precursors for the synthesis of the desired mRNA molecule by T7, SP6, or T3 RNA polymerase. In addition to the linearized DNA template and RNA polymerase, the IVT reaction also requires other components: ribonuclease inhibitor, pyrophosphatase, polymerase cofactor MgCl_2_, and a pH buffer containing polyamine and antioxidants.^451^ After a few hours of IVT, milligram quantities of the desired mRNA can be produced per milliliter of transcription reaction.^452^ Compared with the traditional production methods for inactivated, subunit, or viral vector vaccines, mRNA vaccine technology avoids complicated and time-consuming production steps, while also reducing the risk of contamination from cell sources.

In addition to the target product, the mRNA IVT reaction has many impurities, such as enzymes, NTPs, DNA, and abnormal transcription products. In a lab setting, treatment with DNase is often used to eliminate DNA from the obtained mRNA preparation, and lithium chloride precipitation is used to purify mRNA further.^115^ Nonetheless, these methods do not enable removal of abnormal transcription products, such as dsRNA and truncated RNA fragments. Abnormal transcription products can activate the host innate immune response, thereby causing inflammation and reducing the translation efficiency of delivered mRNA. Previous studies showed that the protein yield of mRNA purified by reverse phase HPLC increases by 10–1000 times.^156,453^ In addition to HPLC, magnetic beads, anion exchange, ultrafiltration, and dialysis can also be used as purification methods. Then, purified mRNA is dissolved in aqueous phase.

Purified mRNA is not suitable for clinical use unless it is protected by a delivery formulation. Most advanced mRNA vaccines employ LNPs as delivery systems. The four lipids (ionizable lipid, DSPC, cholesterol, and PEG-lipid) constituting LNPs are dissolved in ethanol, and each solution is prepared at a known concentration, then mixed at different molar ratios to prepare the mixed lipid phase. The target mRNA is then diluted in a buffer solution at pH ~4 to prepare the aqueous phase. With an appropriate lipid/mRNA ratio, the lipid solution is mixed with the mRNA aqueous phase in a microfluidic or T-junction channel to obtain lipid-encapsulated mRNA. The assembly principle underlying the mRNA-LNP preparation is that the ionizable lipid becomes protonated in the aqueous phase at pH 5.5. Due to its positive charge, it electrostatically binds to the negatively charged mRNA, thereby promoting vesicle formation and encapsulation of mRNA by hydrophobic forces. Once vesicles are formed, dilution and subsequent concentration by ultrafiltration are carried out to remove ethanol and replace the buffer to increase the pH of the preparation. At this time, the ionizable lipid adopts a more hydrophobic, uncharged condition, which drives vesicle fusion to promote encapsulation of mRNA, thereby forming a stable mRNA-LNP spherical complex.^289^ Finally, the obtained mRNA vaccine preparation is further submitted to final processing steps of sterile filtration and filling.

### Quality control of mRNA vaccines

Although the mRNA vaccine is a new technology and its production relatively simple for scaling-up, most approaches employed during the process are sophisticated, thus quality control of mRNA vaccine production still represents a challenge.^454^ Safety, efficacy, and quality control of vaccine production are determined by measuring critical process parameters (CPPs) and intermediate critical quality attributes (IQAs). The management of LNPs encapsulation is directly related to the quality of the final mRNA vaccine, particularly involving target gene sequence design, raw materials, mRNA purity and integrity, and mRNA/lipid ratio. Quality control of mRNA vaccines should adhere to criteria preconized by laws and regulations of the producing countries. Quality control and quality management should be incorporated throughout the production process and life cycle of mRNA vaccines, thereby submitting the entire chain to stringent quality control monitoring.^455–464^ Quality control of COVID-19 mRNA vaccines focuses on raw materials (plasmids, biobanks, lipids, nucleotides, and enzymes used in the production process), semi-finished, and final product.^445,465^

Each country may have different regulamentations depending on whether the plasmid is used as a raw material for the production of mRNA vaccines. However, plasmid quality control must be under constant vigilance, i.e., ensuring the target gene sequence is unmodified, therefore the production process must be tightly controlled. Other biological features of plasmids, such as purity, proportion of supercoiled structures, and residual substance derived from plasmid extraction, should also be considered. In addition to strain quality control, ensuring that bacteria employed in transformations are indeed *Escherichia coli*, plasmid gene sequence, retention rate, copy number, and bacterial strain purity are parameters that should also be considered.

Lipid excipients are essential in mRNA vaccine production. Lipid functions should be assessed in addition to their source, physical and chemical features, such as appearance, type, purity, and residual solvents. Moreover, evaluation with pharmaceutical drugs should also be performed to ensure that novel lipid excipients are in compliance with applicable regulations. Furthermore, any animal (including human)-derived starting or raw material should be submitted to control to determine the source, quality control, and risk assessment, as well as should comply with WHO guidelines on transmissible spongiform encephalopathies concerning biological and pharmaceutical products.^466^

In addition, identification, purity, content of the mRNA stock solution must be considered. Process impurities in the drug substance include residual DNA, RNA polymerase, restriction endonuclease, DNA ligase, unincorporated nucleotides, caps, dsRNA, and misfolded RNA. Proper mRNA capping plays a critical role in mRNA transcription and translation efficiency, as well as in reducing inflammation response. As a result, determining mRNA capping efficiency is critical for quality control of mRNA vaccines, which is usually determined by liquid chromatography and mass spectrometry (LC-MS).^467^

Lipid-encapsulated mRNA forms the mRNA-LNP complex, which is a critical step in mRNA vaccine technology. A reproducible production process should be guaranteed by ensuring proper calibration and adjustment of encapsulating pump equipment and flow rate accuracy after a comprehensive and in-depth optimization. To assure size and homogeneity of LNPs, appropriate criteria should be stipulated, which in turn can increase vaccine stability and induce a better immune response. As a result, mRNA-LNP intermediate products must be scrutinized by measuring average particle size, particle size distribution, mRNA content, encapsulation efficiency, and sterility.

Collectively, it is paramount that internal quality control and release standards must be prepared. The mRNA vaccine should be stored and transported under ultra-low temperature conditions, due to the intrinsic instability of mRNA. Onpattro™, the first approved siRNA drug, also relies on an LNP-based delivery system,^308^ and the drug can be stored at 2–8 °C for three years. Studies showed that mRNA is highly susceptible to degradation by the ubiquitous RNases, therefore ultra-low temperature storage is recommended to limit RNase activity whilst ensuring mRNA stability effectively. In addition, ultra-low temperature preservation is more conducive to maintaining the original conformation of mRNA-LNP complexes.^468^ In fact, mRNA vaccines that have been licensed for clinical trials, commercialization, or emergency use must currently be stored and delivered at low temperatures. Although the current delivery system used in mRNA vaccines is based on LNPs, storage conditions and stability vary among vaccine manufacturers. For instance, the mRNA-1273 vaccine from Moderna maintains its effectiveness for six months at −20 °C, whereas the effectiveness of the BNT162b2 vaccine from BioNTech is maintained for six months when stored at −60 to −80 °C. Conversely, the CVnCoV vaccine from CureVac can be stored at −60 °C for three months. Therefore, existing COVID-19 mRNA vaccines undoubtedly require storage under ultra-low temperatures.^469^ As a result, appropriate verification of cold chain transportation and storage conditions should be taken into consideration in order to ensure the quality of the mRNA vaccine.^470,471^ A more recent storage approach involves employing freeze-drying to mRNA vaccine conservation, which was shown to effectively reduce storage requirements, being thus considered a promising technique to improve mRNA vaccine stability.^472,473^

Furthermore, for scientific and ethical standards, the international pharmacopeia and WHO urged the reduction of animal trials, and instead employing appropriate in vitro alternative approaches for evaluating mRNA safety and efficacy.^474^ At present, with the expansion of COVID-19 mRNA vaccine production and the incorporation of new production lines, changes in the production process have become inevitable. It is necessary to perform inter-batch quality comparisons based on relevant regulations and standards, including procedures and data requirements preconized in WHO guidelines.^475^ If quality comparison studies cannot fully attest product safety, effectiveness, and controllability, bridging studies in clinical trials are likely to offer a more reliable perspective.

## Conclusion and future perspectives

After over 30 years of research, mRNA vaccines have become a promising technology platform for vaccine development. Prior to the emergence of COVID-19, mRNA technology was mostly used for developing novel cancer therapeutic drugs showing promising results. The COVID-19 pandemic has fostered the growth of mRNA vaccine platforms as a means to prevent and treat several infectious diseases, and a new generation of vaccines has progressively reached the public and gained increasing attention. At the moment, COVID-19 mRNA vaccines are playing a key role in limiting the spread of the current pandemic. mRNA vaccines, unlike traditional vaccines, may enable adjustment of antigen design and even allow combining sequences from several variants to respond to new mutations in the virus genome. In the future, the mRNA technology platform will enable preventing and managing infectious diseases as well as treating other disorders. Due to its advantages, such as a quick development cycle, no requirement for cell culture, and high immunogenicity, an mRNA vaccine has become the world’s first COVID-19 vaccine authorized by the FDA. However, the requirement for storage at ultra-low temperature conditions might represent a challenge in transportation and storage of mRNA vaccines. Therefore, stability of mRNA vaccines has to be further explored and optimized. Moreover, delivery of mRNA has evolved from its naked form to LNP-based delivery. Additional studies are being conducted to explore novel polymer materials to be used in mRNA delivery in the future. We believe that the safety and protection conferred by current mRNA vaccines are satisfactory, but their long-term protection will only be determined after additional clinical studies are performed.
